# High-throughput drug screening identifies EGFR/MAPK pathway targeting sensitivities in organoid models of ovarian carcinosarcoma

**DOI:** 10.1186/s13046-025-03629-8

**Published:** 2026-01-06

**Authors:** Andrew Farrell, Genevieve Dall, Cassandra J. Vandenberg, Kristy Shield-Artin, Elizabeth L. Kyran, Tim Blackmore, Ratana Lim, Rachael Taylor, Chloe Neagle, Gayanie Ratnayake, Tao Tan, Dmitri Mouradov, Anthony Hadla, Kate Jarman, Sally Beard, Andrew Jarratt, Jocelyn S. Penington, Matthew J. Wakefield, Anthony T. Papenfuss, Clare L. Scott, Holly E. Barker

**Affiliations:** 1 The Walter and Eliza Hall Institute of Medical Research, Parkville, VIC 3052 Australia; 2 Department of Medical Biology, University of Melbourne, Parkville, VIC 3010 Australia; 3 Cancer Research UK Cambridge Institute, Cambridge, CB2 0RE UK; 4 The Royal Women’s Hospital, Parkville, VIC 3052 Australia; 5 Department of Obstetrics and Gynaecology, University of Melbourne, Parkville, VIC 3010 Australia; 6 Peter MacCallum Cancer Centre, Victorian Comprehensive Cancer Centre, 305 Grattan Street, Melbourne, VIC 3000 Australia; 7 Sir Peter MacCallum Department of Oncology, University of Melbourne, Parkville, VIC 3010 Australia; 8 Cancer Biology and Stem Cells Division, The Walter and Eliza Hall Institute of Cancer Research, 1G Royal Pde, Parkville, VIC 3052 Australia

**Keywords:** Ovarian carcinosarcoma, Epithelial-to-mesenchymal transition (EMT), N-MYC, EGFR, MAPK pathway, Organoids, Eribulin, Cisplatin, Combination therapy

## Abstract

**Background:**

Ovarian carcinosarcoma (OCS) is a rare and aggressive tumour type with limited treatment options. Standard therapy includes platinum agents, but responses are poor. OCS highly express mesenchymal markers, such as N-MYC and HMGA2. The microtubule-targeting drug eribulin can reduce expression of N-MYC and HMGA2 in OCS PDX models and functionally reverse EMT in OCS cell lines.

**Methods:**

In this study, we carried out drug screens in the presence of cisplatin or eribulin to identify synergistic combinations. We validated top combinations in our unique OCS cell line, organoid and PDX models.

**Results:**

The most effective combination treatments in OCS organoid models involved eribulin, whereas cisplatin-based combination therapies were more effective in high-grade serous ovarian cancer (HGSOC) models. Eribulin combined with either an EGFR inhibitor (erlotinib) or a MEK inhibitor (mirdametinib/PD0325901) were the most effective combinations in OCS models, with a synergistic effect being observed in two (out of four) models for each combination. Mechanistically, OCS models appeared to be particularly reliant on EGFR and MAPK signalling in vitro, especially in tumours with *TP53* mutation. In vivo, only modest improvements in survival were observed for eribulin plus erlotinib, however, two of the three OCS PDX models tested were found to have drug resistance mechanisms, such as high *ABCB1* expression (encoding the multi-drug resistance protein which causes drug efflux) or a KRAS constitutive activation mutation (a known resistance mechanism to EGFR inhibitors). KRAS mutant OCS cell lines and organoids were sensitive to dual targeting of the EGFR/MAPK pathway, with statistically greater synergy observed when eribulin was added as a third drug.

**Conclusions:**

OCS is the most aggressive, drug-resistant gynaecological malignancy and eribulin-based combination therapies, particularly triple combination therapies, have the potential to improve patient outcomes.

**Supplementary Information:**

The online version contains supplementary material available at 10.1186/s13046-025-03629-8.

## Introduction

Ovarian carcinosarcoma (OCS), also known as malignant mixed Müllerian tumour, accounts for 1–4% of ovarian malignancies [1, 2], and is a highly aggressive, heterogeneous cancer with poor prognosis [3, 4]. OCS is biphasic, containing both epithelial (carcinoma) and mesenchymal (sarcoma) components [2]. Molecular analyses have indicated that OCS are monoclonal [5–11], and we have recently also shown that they are most likely to arise by the conversion theory, where the carcinoma undergoes epithelial-to-mesenchymal transition (EMT) to form the sarcomatous component [11, 12].

We have previously shown that upregulation of the N-MYC/LIN28B pathway is a common feature of OCS [11]. This is supported by the high expression of another member of this pathway, high-mobility-group AT-hook protein 2 (HMGA2), which has been observed in 60% of OCS cases [13]. Expression of HMGA2 has been observed in many other cancers, in comparison to normal adult tissues, which lack HMGA2 expression [14]. HMGA2 has been shown to induce EMT through multiple mechanisms, including through activation of TGF-β signalling and regulating E-cadherin repression, and is associated with metastasis in many cancers, such as oral squamous cell carcinoma, pancreatic ductal adenocarcinoma, and colorectal cancer [15–22]. Expression of N-MYC has also been associated with therapy resistance in multiple cancer types [23–25]. We recently demonstrated that the microtubule-targeting drug eribulin, which had previously been shown to reverse EMT [26], successfully inhibited the growth of OCS and reduced the expression of N-MYC and HMGA2 in these tumours [11]. Eribulin is now being trialled in combination with pembrolizumab for individuals with OCS or uterine carcinosarcoma (NCT05619913).

With limited alternatives currently available, treatment strategies for OCS in the clinic are the same as those proven to be most effective for the more prevalent subtype of ovarian cancer, high-grade serous ovarian cancer (HGSOC). This involves cytoreductive surgery and platinum-based chemotherapy [2, 27]. However, OCS are more chemoresistant than HGSOC, likely due to the sarcomatous component, and up-regulation of N-MYC and HMGA2, resulting in poor treatment response [4, 28, 29]. Furthermore, most patients relapse within one year of treatment and the median overall survival is less than two years [29]. Therefore, better treatment strategies are desperately needed for individuals diagnosed with this aggressive cancer.

In this study, we used our unique OCS cell line, organoid and patient-derived xenograft (PDX) models to investigate responses to a range of drugs, with a focus on those that could potentially target EMT or N-MYC. Due to the ability of eribulin to inhibit EMT, reduce N-MYC and HMGA2 expression, and reduce tumour growth in our preclinical models of OCS [11], we investigated eribulin-based combination therapies to improve response. In addition, we investigated cisplatin-based combination therapies, to potentially enhance responses to the current standard treatment, in order to improve the prognosis of OCS patients. Finally, a rational triple combination was investigated as a potential therapeutic strategy for this aggressive malignancy.

## Materials and methods

### Patient samples and generation of patient-derived xenograft (PDX) models

Tumour tissue (both fresh and formalin-fixed paraffin-embedded [FFPE]) and blood samples were obtained for patients who provided informed consent to the Stafford Fox Rare Cancer Program (SFRCP) at The Walter and Eliza Hall Institute of Medical Research (WEHI, Melbourne, Australia). The SFRCP was approved by the Melbourne Health Human Research Ethics Committee, project #2015.300, with additional approval from WEHI HREC, G16/02. All experiments involving animals were performed according to the Australian Code for the Care and Use of Animals for Scientific Purposes 8th Edition (updated 2021) and were approved by the WEHI Animal Ethics Committee (2019.024 and 2022.030). PDX #013, #105, #201, #217 and #233 have been previously described [11, 30–33]. PDX #264 was established from tumour tissue obtained from patients consented to the SFRCP. PDX PH003, PH142 and PH419 were obtained from the Mayo Clinic and established as previously described [11]. Fragments of tumour tissue were transplanted subcutaneously into NOD/SCID/IL2Rγnull recipient mice (T1 = passage 1). Harvested tumours were histologically assessed by a gynaecological pathologist, using sections stained with H&E, pan-cytokeratin and vimentin, to ensure PDX models resembled the original patient tumours.

### Generation and culturing of cell lines

The mouse OCS (genetically-engineered mouse model [GEMM]) cell line, and human OCS cell lines PH142 and PH419 were generated as previously described [11]. The PH003 and #105 cell lines were generated from T3 (3rd passage) and T5 (5th passage) PDX tumours, respectively. Briefly, tumours were manually minced into a slurry, digested with collagenase (Sigma), dispase (Thermo Fisher Scientific), and DNase (Sigma), and filtered through a 100 μm cell strainer (Bio-Strategy, FAL 352350). Cells were initially plated in Primaria flasks (Corning) and cultured in growth media for at least 10 passages. Cell identity was confirmed by short tandem repeat (STR) profiling and whole genome sequencing (WGS). PH003 and PH419 cell lines were maintained in FV media as previously described [11]. For the culture of PH142 and #105 cell lines, 2D media was used: Advanced DMEM/F-12 (Gibco), 1X penicillin-streptomycin (Gibco), 1X N2 Supplement (Gibco), 10% fetal bovine serum (FBS; Thermo Fisher), 50 ng/ml basic FGF (Peprotech), 20 ng/ml hEGF (Sigma), 10 ng/ml LONG R3 IGF1 (Sigma), 2 µg/ml heparin (Sigma), 36 ng/ml hydrocortisone (Sigma), 0.5 ng/ml beta-estradiol (Sigma) and 5 µg/ml insulin (Roche). OVCAR8 and PEO4 HGSOC cell lines were obtained from the NCI, validated by STR profiling, and cultured in FV media. Cell lines were routinely passaged using 1X Trypsin-EDTA and were used at consistent passages and growth rates for relevant assays.

### Generation and culturing of organoids

All organoid models were generated from vehicle or untreated PDX tumours. Tumours were digested as above and cells washed and counted. For immunohistochemistry (IHC), cells were resuspended in 100% Matrigel (Bio-strategy) and four 10 µl droplets plated per well of a 24-well non-treated plate (Thermo Fisher Scientific) at a density of 10,000–20,000 cells per droplet. Plates were incubated at 37 °C for 10 min to allow Matrigel to solidify before adding 3D growth media. After 1–2 weeks of growth (with regular growth media changes), organoids were collected, washed, resuspended in HistoGel (Thermo Fisher Scientific), fixed in 10% formalin and blocked in paraffin for IHC analysis. For drug assays, cells were resuspended in 3D media containing 5% Matrigel at a density of 4,000 cells per well in 384-well clear bottom non-treated plates (Nunc). Organoids were cultured for 3–5 days before proceeding to drug assays. 3D media was comprised of a modified version of previously described organoid media [34] containing Advanced DMEM/F-12, 1X GlutaMAX (Gibco), 10 mM HEPES (Gibco), 1X penicillin-streptomycin, 10 ng/ml hEGF, 500 ng/ml hydrocortisone, 100 nM beta-estradiol, 100 ng/ml FGF10 (Peprotech), 1 ng/ml Wnt3a (Peprotech), 100 µg/ml Primocin (InvivoGen), 10 µM forskolin (Tocris), 0.5 µM A83-01 (Tocris), 2% B-27 Supplement (Invitrogen), 1 mM nicotinamide (Sigma), 1.25 mM N-acetylcysteine (Sigma) and 10 µM Y27632-hydrochloride (Sigma-Aldrich). Fresh and viably frozen digested tumours were routinely used for organoid growth and drug assays were only performed when optimal organoid growth was observed.

### DNA sequencing and data analysis

DNA extracted from FFPE tumour and fresh blood patient samples was submitted to the Victorian Clinical Genetics Services (VCGS) for whole exome sequencing (WES). DNA extracted from fresh tumour and blood patient samples was submitted to the University of Melbourne Centre for Cancer Research, Australia, for whole genome sequencing (WGS). For tumours where matched blood samples were not available, DNA was submitted to the Molecular Oncology Laboratory at Peter MacCallum Cancer Centre for analysis using the Illumina TruSight Oncology 500 Assay (TSO500) or Molecular Screening and Therapeutics Panel (MoST; together referred to as “Cancer Panel” in Fig. 2). Additional sequencing was carried out where possible using the next generation sequencing (NGS)-based BROCA-HR assays as previously described [30]. DNA extracted from the PH003, PH142, PH419 and #105 cell lines was submitted to VCGS for WGS and DNA extracted from #233 organoids was submitted to VCGS for WES. All sequencing was completed to an average depth of 30X. Sequencing data was processed and analysed as previously described [11].

### Drug library screens

The GEMM cell line was used to screen a library of 3,885 compounds with FDA-approval and/or proven preclinical activity, alone and in combination with cisplatin or eribulin. The library was constructed from four known drug libraries: LOPAC^®^ (1280 compounds), Prestwick (1200 compounds), Selleck (108 compounds) and Tocris (1118 compounds). A few compounds were present in more than one library. The library also included 179 in-house targeted agents. To determine EC20 values for cisplatin and eribulin, GEMM cells were plated in 384-well PS White µClear^®^ microplates (Interpath) and treated with 10-point dose ranges of cisplatin or eribulin for 72 h. Cell viability was measured using CellTiter-Glo^®^ (Promega) as per manufacturer’s instructions, with luminescence quantified using a CLARIOstar Plus machine (BMG Labtech). All values were normalised to values obtained from control (untreated) wells. Concentrations of each drug that killed 20% of cells (i.e. EC20 values) were calculated from non-linear fit curves. For the drug screen, cells were plated in 384-well plates containing single doses (1 µM) of each compound in the drug library alone or in the presence of EC20 concentrations of cisplatin (650 nM) or eribulin (20 nM) and cultured for 72 h. Cell viability was measured as described previously with values normalised to values obtained from control wells (containing cells exposed to DMSO only). Delta (Δ) effect values were calculated by subtracting the effect (% cells killed) of each compound alone from the effect of each compound when combined with cisplatin or eribulin.

181 compounds were selected for validation screens (with seven drugs run in duplicate or triplicate). GEMM cells were plated in 384-well plates containing 5-point dose ranges (1 in 3 dilutions from 3 µM down to 37 nM) of each compound alone or in the presence of EC20 concentrations of cisplatin or eribulin and cultured for 72 h. Cell viability was measured as described previously with values normalised to values obtained from control wells (containing cells exposed to DMSO only). ΔAUC (area under the curve) values were calculated by subtracting the AUC for each compound alone from the AUC for each compound when combined with cisplatin or eribulin.

### Drug assays in cell lines and organoids

Master drug plates were created in 384-well U-bottom microplates (Revvity) using a JANUS liquid handling robot (PerkinElmer). For analysis of single agents, 10-point doses of drugs dissolved in DMSO were plated with 1 in 5 titrations from left to right. For analysis of combinations, 5- or 6-point doses of drugs dissolved in DMSO were plated with 1 in 5 titrations from left to right on one plate and top to bottom on a second plate for the cisplatin and eribulin combination drugs. This resulted in a matrix of all possible combinations of drug concentrations once added to the cells or organoids. To test for triple combinations, a third drug, eribulin was added to each plate at a dosage of 0.1 nM. Cisplatin required manual addition due to insolubility in DMSO. DMSO 0.5% (v/v) and 10 µM bortezomib served as negative (vehicle) and positive (killing) controls, respectively. Cell lines were plated in 384-well PS White µClear^®^ microplates at 2,000 cells per well. Organoids were plated in 384-well clear bottom microplates at 4,000 cells per well as previously described. Drugs were added to cells (from master drug plates) on day 2 and organoids on days 3–6 (depending on organoid growth rate) using the JANUS liquid handling robot. Cell and organoid viability were measured as previously described at 72 h or 7 days after adding drugs, respectively. Viability was normalised to viability measured in control (untreated) wells, and where applicable, synergy outputs were generated using the SynergyFinder + R package (available online at synergyfinder.org) [35]. Synergy and sensitivity scores were calculated with full baseline correction and per biological replicate, followed by calculation of mean synergy and sensitivity scores across biological replicates by pooling normalised data.

### Imaging of organoids

Where possible, organoid cultures were imaged daily for 7 days by bright-field microscopy on an automated Nikon Eclipse Ti2 Inverted Microscope System (NIS-Elements AR software version 5.21.03, 64-bit). Image analysis to determine organoid size and viability was performed using ImageJ software, as described previously [36]. Imaging data was also used to exclude possible outliers in the data as necessary and to inform on the optimal growth rate point for drugging and CTG endpoint analysis.

### Immunohistochemistry

FFPE from cell line, organoid and PDX samples were sectioned and stained with haematoxylin and eosin (H&E) or the following antibodies: anti-Ki67 (mouse: D3B5, Cell Signalling; human: MIB-1, Dako), anti-PAX8 (Proteintech #10336-1-AP), anti-p53 (mouse: CM5, Novacastra; human: DO-7, Dako), anti-pan cytokeratin (panCK; mouse: polyclonal, Abcam; human: AE1/3, Dako), anti-vimentin (Cell Signaling Technology #5741), anti-HMGA2 (Cell Signaling Technology #8179). H&E and IHC slides were scanned digitally at 20x magnification using the Pannoramic SCAN II (3DHISTECH Ltd.).

### Western blotting

Western Blotting was performed as described previously [11]. Membranes were probed with antibodies specific for ERK, pERK, CDK1, pCDK1, EGFR (Cell Signaling Technology, Cat #9102, #9101, #9116, #9111, #4267), ɣ-H2A.X (Abcam, ab22551), p-Histone H3 (Millipore, Cat #06–570) and β-actin (Sigma-Aldrich Cat# A5441). The polyclonal antibody for Hsp70 was made in house.

### Immunofluorescence

Cell lines were plated in 384-well PS White µClear^®^ microplates at 5,000 cells per well. The following day, cells were treated with EC50 concentrations (concentrations that kill 50% of cells) of cisplatin or eribulin, and/or combination drugs. After 24 h, the cells were fixed with 4% paraformaldehyde (Sigma) and washed with Dulbecco’s Phosphate Buffered Saline (DPBS; Gibco). Cells were then permeabilised with 0.2% Triton X-100, washed, and blocked with 1% bovine serum albumin (BSA; Sigma)/2% FBS in DPBS before being stained with γ-H2A.X-Alexa-647 antibody (Millipore; 1:250 in 2% BSA, 1% FBS, DPBS) and counterstained with Hoechst 33,342 stain (Thermo Fisher). The plates were then imaged using an Opera Phenix Plus (Revvity) at 40x water immersion and images processed using Columbus analysis tools to determine numbers of pan-γ-H2AX cells, γ-H2AX foci, micronuclei as well as enlarged, undivided (≥4 N) nuclei. Total genotoxic events were determined by the sum of all γ-H2AX-positive cells, micronuclei and 4 N nuclei.

### In vivo studies

Recipient mice bearing T2-T7 PDX tumours (180–300 mm^3^ in size) were randomly assigned to cisplatin (Pfizer), eribulin mesylate (Halaven, Eisai), ZN-c3 (E1000, SelleckChem), ganetespib (HY-15205, MedChemExpress), mirdametinib (S1036, SelleckChem), erlotinib (HY-50896, MedChemExpress), or vehicle treatment groups. In vivo cisplatin treatments were performed by intraperitoneal (IP) injection of 3 or 4 mg/kg, on days 1, 8 and 18. The regimen for eribulin treatment was by IP injection once only at 0.5 mg/kg, or 1–3 times a week for three weeks at 0.5 mg/kg, depending on the model being treated. The regimen for mirdametinib was oral gavage (OG) 5 days on/2 days off for 3 weeks, at 3 mg/kg. The regimen for erlotinib was OG 5 days on/2 days off for 3 weeks, at 100 mg/kg. The regimen for ganetespib was intravenous (IV) injection, twice weekly for 3 weeks, at 50 mg/kg or 75 mg/kg. The regimen for ZN-c3 was OG 3 days on/4 days off for 3 weeks, at 30 mg/kg. The vehicle for cisplatin and eribulin treatment was DPBS. For other drug formulations see Table 1. Data collection was conducted using the Studylog LIMS software (Studylog Systems, San Francisco). Graphing and statistical analysis was conducted using the SurvivalVolume package [37]. Harvested PDX tumours were histologically assessed by a gynaecological pathologist, using sections stained with H&E, pan-cytokeratin and vimentin, to assess composition of carcinoma and sarcoma components.

**Table 1:** Drug treatments for *in vivo* studies

Drug	Formulation	Dose (mg/kg)	Route of delivery	Dose volume (µL/g)	3-week treatment regimen
Cisplatin	DPBS	4	IP	20	D1, D8, D18
Eribulin	DPBS	0.5	IP	10	single dose
Eribulin	DPBS	0.5	IP	10	weekly
Erlotinib	10% DMSO, 40% PEG300, 5% Tween80 and 45% ddH2O	100	OG	10	5 days on/2 days off
Ganetespib	10% DMSO, 18% Cremophor RH 40, 3.6% dextrose and 68.4% ddH2O	50	IV	10	twice weekly
Mirdametinib	5% DMSO/40% PEG300/5% Tween80/50% ddH2O	3	OG	10	5 days on/2 days off
ZN-c3	20% 2-hydroxypropyl-beta-cyclodextrin	30	OG	10	3 days on/4 days off

A 3-week treatment regimen was used for all drugs and combinations (except single dose eribulin).

For in vivo treatments, Halaven, the clinical preparation of eribulin mesylate, was used.

### Statistical analysis

Data were analysed using matched multiple-comparisons one or two-way ANOVA unless otherwise stated and considered significant when the *p* value was < 0.05. Bar graphs represent the mean and standard error across independent experimental repeats (at least *n* = 3) unless otherwise stated. Survival analysis was performed using the log rank test on Kaplan-Meier survival function estimates. Statistical significance representations: *p < 0.05, **p < 0.01, ***p < 0.001, ****p < 0.0001.

## Results

### Drug screening of a murine OCS cell line in the presence of either cisplatin or eribulin identified synergies involving independent drug classes

**Fig. 1:**
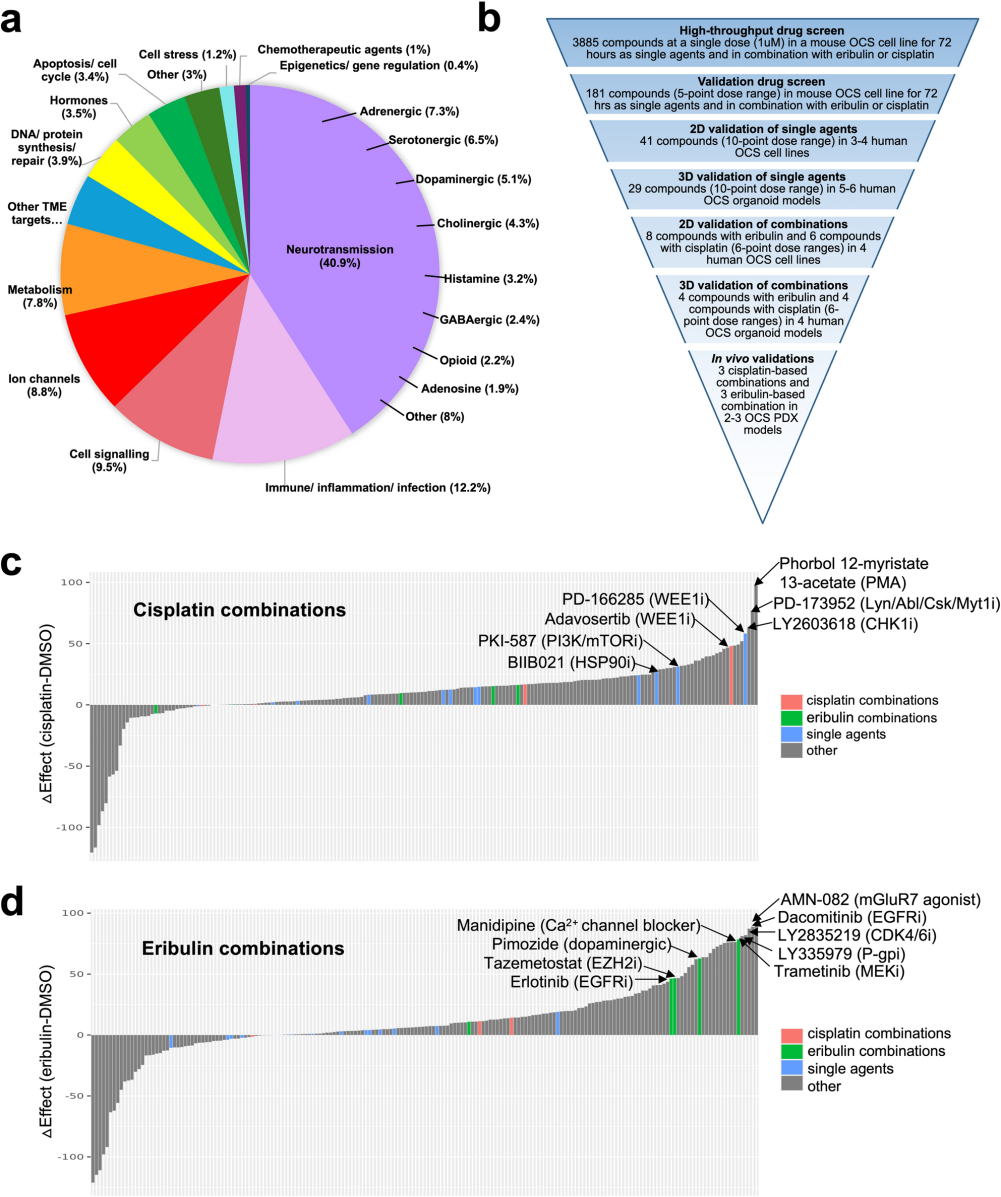
Drug screening of a mouse OCS cell line identifies sensitivity to independent cisplatin- and eribulin-based combinations; (**a**) Breakdown of drug classes included in the initial drug screen involving 3,885 compounds; (**b**) Overview of the drug screening process from the initial drug screen of 3,885 compounds down to the selected 2–3 combinations for both cisplatin and eribulin tested in vivo; (**c**) Waterfall plot of cisplatin-based combination drug effects in the validation screen. ΔAUC values are calculated by subtracting the AUC for each compound alone from the AUC for each compound in combination with cisplatin. The most effective combinations are indicated as well as specific cisplatin-based (red), eribulin-based (green) and single agents (blue) that were selected for further validation; (**d**) Waterfall plot of eribulin-based combination drug effects in the validation screen. ΔAUC values are calculated by subtracting the AUC for each compound alone from the AUC for each compound in combination with eribulin. The most effective combinations are indicated as well as specific cisplatin-based (red), eribulin-based (green) and single agents (blue) that were selected for further validation; AUC, area under the curve

We have previously described a murine OCS cell line, which we derived from a tumour arising in a genetically-engineered mouse model (GEMM), with p53 inhibition and LIN28B over-expression in fallopian tube secretory epithelial cells [11]. This GEMM cell line was used in a drug screen involving 3,885 compounds with FDA-approval or proven preclinical efficacy (Fig. 1a). The overall process of drug screening and in vitro and in vivo validations is summarised in Fig. 1b. The drug screen was also carried out in the presence of either cisplatin, as the current standard therapy for OCS is platinum-based, or eribulin, which is being tested in a clinical trial for OCS (NCT05619913) as a result of our previous work [11]. EC20 concentrations were determined for cisplatin (650 nM) and eribulin (20 nM) in the GEMM cell line to ensure significantly increased cell killing could be observed in the drug screens relative to this concentration (Supplementary Figure S1a).

Each compound was screened at a concentration of 1 µM, alone or in combination with 650 nM cisplatin or 20 nM eribulin (i.e. EC20 concentrations). GEMM cells demonstrated sensitivity to compounds with broad activities, such as proteasome inhibitors, antimetabolites and topoisomerase inhibitors. GEMM cells also demonstrated sensitivity to compounds with more specific activities, such as inhibitors of the non-receptor tyrosine kinase SRC, heat-shock protein 90 (HSP90) and cyclin-dependent kinases (CDKs) (Supplementary Figure S1b, Supplementary Table S1). Enhanced cell killing in the presence of cisplatin was observed for drugs that inhibit cell-cycle checkpoint proteins (i.e. CHK1 and WEE1), HSP90 and CDKs (Supplementary Figure S1c, Supplementary Table S2). Enhanced cell killing in the presence of eribulin was observed for drugs that inhibit the epidermal growth factor receptor (EGFR) (Supplementary Figure S1d, Supplementary Table S3).

From the initial 3,885 compounds, 181 of the most effective compounds were selected for validation screens based on having potential roles in inhibiting EMT or N-MYC, or displaying enhanced cell killing in the presence of cisplatin or eribulin. The GEMM cells were treated with these 181 compounds at a 5-point dose range (3 µM to 37 nM) alone and in combination with 650 nM cisplatin or 20 nM eribulin. Strong single agent responses again included proteasome inhibitors, antimetabolites, topoisomerase inhibitors, HSP90 inhibitors (HSP90i) and CDK inhibitors (CDKi) (Supplementary Table S4). Enhanced cell killing in the presence of cisplatin was again observed for CHK1 and WEE1 inhibitors, HSP90i, CDKi, as well as inhibitors of the PI3K/AKT/MTOR pathway, SRC family kinases, and the mitogen-activated protein kinase pathway (MAPK), such as MEK inhibitors (MEKi) (Fig. 1c, Supplementary Table S5). Enhanced cell killing in the presence of eribulin was again observed for EGFR inhibitors (EGFRi), as well as CDKi, MEKi, an EZH2 inhibitor (EZH2i) and calcium channel blockers (Fig. 1d, Supplementary Table S6).

### Validation of single agent hits in human OCS cell lines, with a focus on drugs targeting EMT and N-MYC, identified specific sensitivities to inhibitors of the proteasome and HSP90

**Fig. 2:**
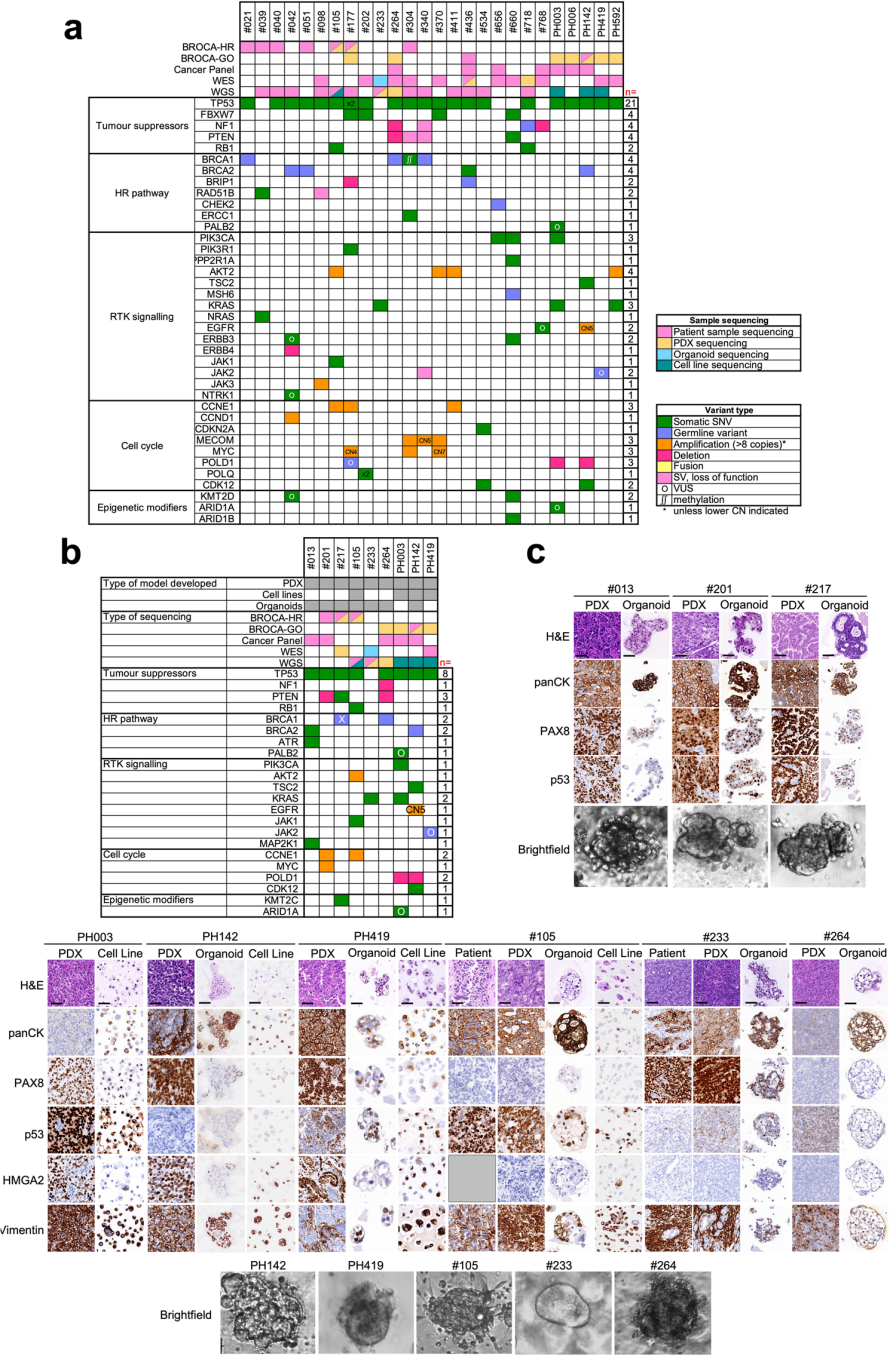
Characterisation of preclinical models used for validation of drug screen;** (a)** The genomic landscape for 21 OCS cases in our Rare Cancer Program and five OCS cases from the Mayo Clinic (with prefix “PH”). The different sequencing methods are indicated at the top. **(b)** OCS preclinical models and their molecular profiles. Types of preclinical models and sequencing methods for patient samples and preclinical models are indicated at the top; **(c)** IHC of samples collected from HGSOC and OCS preclinical models and patient samples, where available. The three HGSOC models are indicated at the top, stained with H&E, panCK, PAX8 and p53. The six OCS models are indicated at the bottom, stained with H&E, panCK, PAX8, p53, HMGA2 and vimentin. Brightfield images of organoids are also shown. WES, whole exome sequencing; WGS, whole genome sequencing; OCS, ovarian carcinosarcoma; HGSOC, high-grade serous ovarian cancer; IHC, immunohistochemistry; H&E, haematoxylin and eosin; panCK, pan-cytokeratin. Scale bar = 50 μm

To assist with selection of compounds to be validated in our human preclinical models, we first molecularly profiled the OCS cases and preclinical models in our Rare Cancer Program. Tumour and blood samples (where available) from 26 individuals diagnosed with OCS were analysed by whole genome sequencing (WGS), whole exome sequencing (WES) or the TruSight Oncology 500 (TSO500™) and/or BROCA panels). The most frequently altered gene was *TP53* (21/26) of cases) (Fig. 2a). Other frequently altered genes included *FBXW7*, *NF1*, *PTEN*, *BRCA1*, *BRCA2* and *AKT2* (4/26 cases each) and *PlK3CA*, *KRAS*, *CCNE1*, *MECOM*, *MYC* and *POLD1* (3/26 cases each) (Fig. 2a). We have successfully derived PDX models from tumour tissue provided for some of these cases (most described previously, including PDXs generated at the Mayo Clinic, USA [11, 30, 33]). We generated an additional OCS PDX model, #264, not previously described. We have successfully derived cell lines and organoid models from some of the PDX models (for some PDX only cell lines could be derived and for others, only organoids were successful). We have previously described the PH142 and PH419 cell lines [11]. We generated two additional human OCS cell lines from PDX models, PH003 and #105. The type of models derived for each case are indicated in Fig. 2b. We characterised the cell lines by WGS, validating that they retained the key driver mutations identified in the original patient tumours (Table 2; Fig. 2b). Molecular profiling indicated that the PI3K/mTOR/AKT pathway, MAPK pathway, and cell cycle checkpoints were potential targets in OCS, given the alterations identified in *PTEN*, *AKT2*, *PIK3CA*, *KRAS*, and *CCNE1* (Fig. 2a + b), which correlates with previous studies (summarised in [38]). Compounds targeting these alterations are included in the validation screens described below. The presence of variants in *BRCA1* or *BRCA2* in two of our OCS models, suggests they may respond to PARP inhibitors (PARPi). PARPi are already approved for the treatment of *BRCA*-mutated ovarian cancer, including OCS [39], therefore, we chose not to include PARPi in our validation experiments. We also characterised the new models by IHC, using pan-cytokeratin (panCK) to define carcinomatous cells, as well as PAX8 and p53 staining in all models, with additional vimentin and HMGA2 staining to define sarcomatous cells in the OCS models (up to 60% of OCS express HMGA2 [13]) (Fig. 2c).

**Table 2:** Molecular profile of HGSOC and OCS models

Model	Histology	Genes with deleterious variants	Amplified genes	Deleted genes	Genes with variants of unknown significance	Models used
OVCAR8	HGSOC	*TP53*,* ERBB2*,* KRAS*,* CTNNB1*	*AKT3*,* CCNE1*,* MYC*			cell line
PEO4	HGSOC	*TP53*,* NF1*,* BRCA2*, BRCA2*				cell line
#013	HGSOC	*TP53*,* BRCA2*,* ATR*,* MAP2K1*				organoids
#201	HGSOC	*TP53*,* BRD4*	*MYC*,* CCNE1*,* BCL2L1*,* SRC*,* AURKA*	*PTEN*		organoids
#217	HGSOC	*TP53*,* PTEN*,* gBRCA1*, BRCA1**,* KMT2C*				organoids
PH003	OCS	*TP53*,* PIK3CA*,* KRAS*			*ARID1A*,* PALB2*	cell line
PH006	OCS	*TP53*	*FGFR1*,* CDK6*	*DNMT3A*		organoids
PH142	OCS	*TP53*,* BRCA2*	*CDK4*,* MDM2*			cell line, organoids, PDX
PH419	OCS	*TP53*			*FGFR1*,* JAK2*	cell line, organoids
#105	OCS	*TP53*,* RB1*,* JAK1*	*AKT2*,* BCL2L1*,* CCNE1*,* FGFR3*,* PLAGL2*,* WHSC1*			cell line, organoids, PDX
#233	OCS	*KRAS*,* SPOP*,* COL2A1*			*HUWE1*	organoids, PDX
#264	OCS	*BRCA1* (germline)			*KIF13A*,* XRCC3*	organoids

*reversion mutation that restores BRCA2 function

**reversion mutation that restores BRCA1 function

We have previously demonstrated that EMT is a key feature of OCS [11]. Therefore, we designed one drug screen validation plate to include inhibitors that have previously been found to block EMT. These included inhibitors of CDK4/SETD8/KMT5A (ryuvidine), HSP90 (AUY922, ganetespib, BIIB021 and BEP800), the proteasome (bortezomib and MG-132), HMG-CoA reductase (simvastatin), SRC/FGFR1/PDGFRβ/WEE1 (PD-166285), and aldehyde dehydrogenase and dopamine beta-hydroxylase (disulfiram, which can also function as a proteasome inhibitor). The response to these compounds was investigated in two human OCS cell lines (PH003 and PH419). To determine whether these compounds specifically targeted EMT, we included two HGSOC cell lines (OVCAR8 and PEO4) for comparison, as these were not expected to harbour features of EMT. The greatest responses (as assessed by lowest EC50 concentrations) were observed for the proteasome inhibitors bortezomib (EC50 range of 0.7–0.9 nM) and MG-132 (EC50 range of 11.0–16.1 nM), the HSP90i BIIB021 (EC50 range of 2.4–44.2 nM) and the SRC/FGFR1/PDGFRβ/WEE1 inhibitor PD-166,285 (EC50 range of 7.2–345.8 nM) (Supplementary Figure S2a, b + c). Despite focusing on drugs predicted to reverse EMT, we did not observe any drugs demonstrating greater activity in OCS cell lines, than that seen in HGSOC cell lines (Supplementary Figure S2d).

A second drug screen validation plate was designed to include potential drugs relevant to N-MYC signalling. These included inhibitors of histone deacetylase (HDAC) (NSC-3852), the PI3K/mTOR/AKT pathway (SF2523 [also inhibits BRD4], fimepinostat [also inhibits HDAC], alpelisib, AZD5363 and BEZ-235), the MAPK pathway (mirdametinib), HMG-CoA reductase (simvastatin, fluvastatin and lovastatin), CDKs (dinaciclib and palbociclib), and the proteasome (ixazomib). The response to these compounds was investigated in all four human OCS cell lines (PH003, PH142, PH419 and #105). The greatest responses (as assessed by lowest EC50 concentrations) were observed for the proteasome inhibitor ixazomib (EC50 range of 1.1–161.7 nM), the PI3K/HDAC inhibitor fimepinostat (EC50 range of 8.5–525.5 nM), the PI3K/mTOR inhibitor PKI-587 (EC50 range 0.8–701.1 nM, with #105 cells being relatively resistant; EC50 of 23.8 µM), and the HMG-CoA reductase inhibitor fluvastatin (EC50 range 70.7–714.6 nM, with PH419 cells being relatively resistant; EC50 of 6.4 µM) (Supplementary Figure S3a, b, c).

### Validation of single agent hits in human OCS organoid models confirmed model-specific sensitivities to inhibitors of the proteasome and HSP90, as well as PI3K and HDAC

**Fig. 3:**
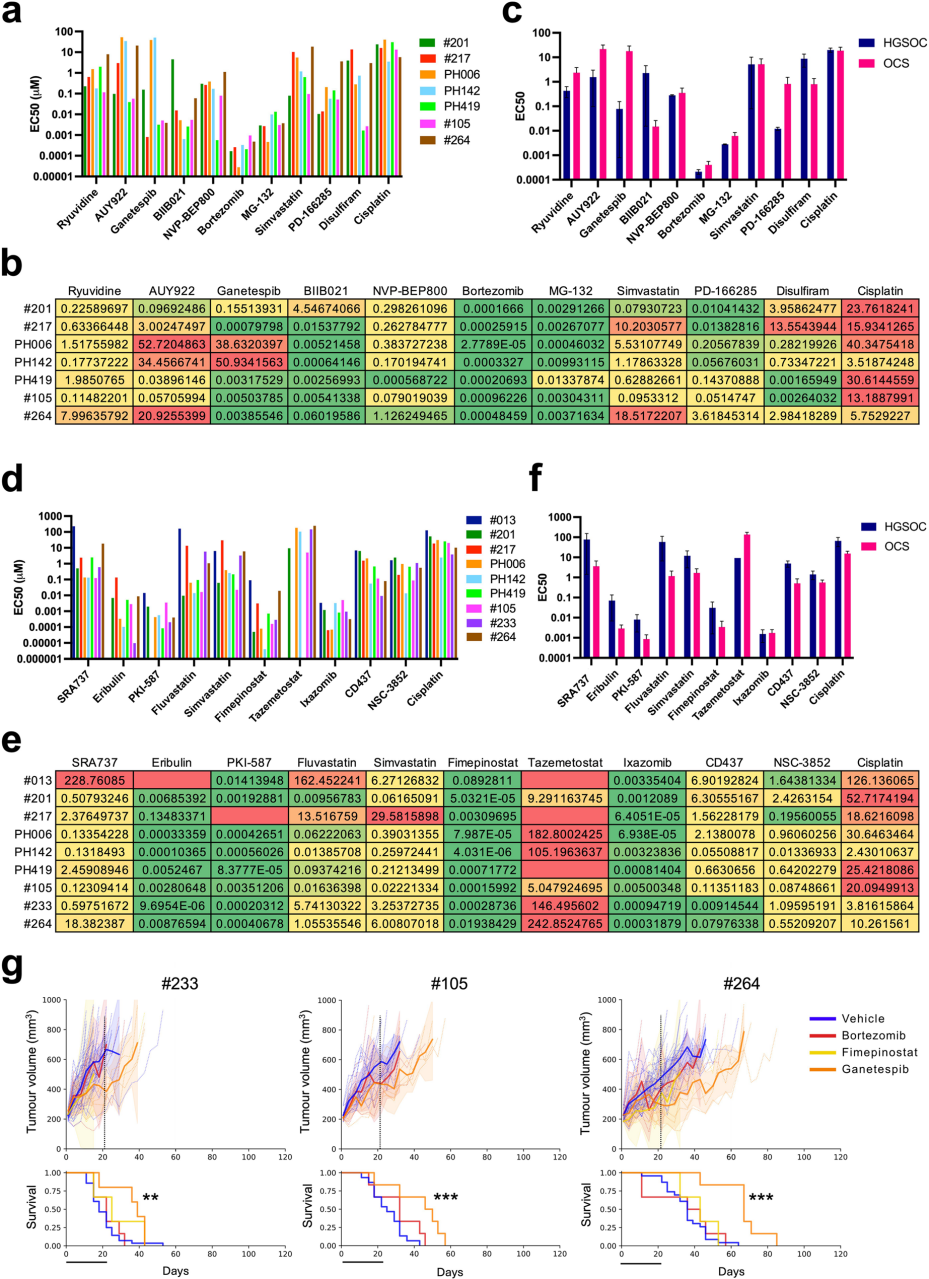
Validation of single agent hits from the drug screen in organoid models; **a**) EC50 values for each of the EMT-related single agents tested in organoid models; b) Heatmap of EC50 values for each EMT-related single agent in each model; **c**) Average EC50 values for the HGSOC models combined compared to OCS models combined; **d**) EC50 values for each of the N-MYC-related single agents tested in organoid models; **e**) Heatmap of EC50 values for each N-MYC-related single agent in each model; **f**) Average EC50 values for the HGSOC models combined compared to OCS models combined; **g**) In vivo treatment of three OCS PDX models with three single agents: fimepinostat, ixazomib and ganetespib. Top graphs show tumour growth over time and bottom graphs are Kaplan-Meier survival curves. **p < 0.01, ***p < 0.001; all comparisons are ganetespib vs vehicle

Organoids are more physiologically relevant models than are cell lines, as they better replicate tumour architecture and drug exposure. Particularly with respect to OCS, organoids are more likely to retain both carcinomatous and sarcomatous components compared to cell lines. Their robustness has been proven in many studies, where they have enabled accurate prediction of patient responses [40]. Therefore, we generated five human organoid models of OCS and two human organoid models of HGSOC from PDX tumour tissue to further validate the most effective compounds identified in the cell lines (Fig. 2b). We characterised these models by IHC, to ensure they resembled the PDX model from which they were derived and maintained both carcinomatous and sarcomatous cells (Fig. 2c). We also validated OCS model #233 grown in 100% Matrigel (for IHC analysis) and 5% Matrigel (for drug screening) by WES, indicating these organoids retained the key driver mutations identified in the original patient tumours (Fig. 2b). We used these models to validate the efficacy of 20 compounds, and compared responses with response to standard therapy of single-agent cisplatin. For drugs relevant to EMT, the greatest responses were again observed for the proteasome inhibitors bortezomib (EC50 range of 0.03–0.96 nM) and MG-132 (EC50 range of 0.46–13.38 nM), and the HSP90i BIIB021 (EC50 range of 0.64–60.20 nM, with #201 being relatively resistant; EC50 of 4.55 µM) (Fig. 3a + b, Supplementary Figure S4a). Again, despite all drugs having a potential role in reversing EMT, only BIIB021 and disulfiram (inhibitor of aldehyde dehydrogenase, dopamine beta-hydroxylase and the proteasome) showed a trend to being more effective in OCS models than HGSOC models (Fig. 3c).

For drugs relevant to N-MYC, the greatest responses were again observed for the proteasome inhibitor ixazomib (EC50 range of 0.06–5.00 nM) and the PI3K/HDAC inhibitor fimepinostat (EC50 range of 4.03 pM – 89.28 nM) (Fig. 3d + e, Supplementary Figure S4b). Good responses were also observed for eribulin (EC50 range of 0.01–134.83 nM, as expected from our previous data [11, 30], with #013 being completely resistant; EC50 not reached) and the PI3K/mTOR inhibitor PKI-587 (EC50 range of 0.08–14.14 nM, with #217 being completely resistant; EC50 not reached) (Fig. 3d + e, Supplementary Figure S4b). A trend was observed for mean EC50 values being lower in OCS models than HGSOC models for 9 of 11 drugs tested (Fig. 3f). This is in keeping with our OCS models generally harbouring higher levels of N-MYC expression compared to our HGSOC models, none of which showed any expression of N-MYC (Supplementary Figure S5).

### The HSP90i ganetespib demonstrated modest improvements in survival in vivo

PDX models maintain cancer heterogeneity to a similar degree as do organoid models, and may replicate therapeutic responses seen in patients more accurately if clinically-relevant dosing is exceeded in vitro [41]. Therefore, we selected three OCS PDX models (#105, #233 and #264) to provide in vivo insights for the most effective compounds identified in the organoid models. Neither bortezomib, ixazomib or fimepinostat treatment had any effect on tumour growth rate or mouse survival (Fig. 3g). While organoids were more sensitive to the HSP90i BIIB021 overall, 5/7 organoid models were also highly sensitive to ganetespib (Fig. 3a + b). Since ganetespib is the most clinically advanced HSP90i with likely an improved safety profile [42], this was the compound selected for in vivo validation. Ganetespib had a modest effect on tumour growth rate (demonstrated by improvements in median time to harvest (TTH) for ganetespib compared to vehicle treatment, which was significant in all models; #105: 50 days vs. 25 days (*p* = 0.0004), #233: 39 days vs. 18 days (*p* = 0.0021), and #264: 67 days vs. 36 days (*p* = 0.0002)) (Fig. 3g).

### Combination drug screening of human OCS cell lines identified novel drug interactions

To identify better therapeutic strategies for OCS, we next validated the most effective cisplatin- and eribulin-based combinations from the drug screen in our human OCS preclinical models. In the initial drug screens, the most effective cisplatin-based combinations involved inhibitors of CHK1, WEE1, HSP90, the PI3K/AKT/MTOR pathway, SRC family kinases, MEK and CDKs (Fig. 1b, Supplementary Table S5). Therefore, we selected the following five drugs for validation with cisplatin in our human OCS cell lines: adavosertib (WEE1i), SRA737 (CHK1i), ganetespib (HSP90i), fimepinostat (PI3K/HDACi) and dinaciclib (CDKi). We also included the combination of cisplatin plus eribulin as this is a combination not previously tested in ovarian cancer that could be clinically relevant. Our hypothesis was that eribulin would reduce the mesenchymal characteristics of OCS cells (which we have observed previously [11]), thus making them more responsive to cisplatin. All drugs involved in the combination analysis and the range of concentrations for each are listed in Supplementary Table S7. We tested these cisplatin-based combinations in all four OCS cell lines; PH003, PH142, PH419 and #105. The highest single agent (HSA) model, where the synergy score quantifies the excess over the highest single drug response [35, 43], was used to analyse drug interaction for each combination. Bliss and ZIP scores are also shown in Supplementary Table S8. All six cisplatin-based combinations were found to be additive (HSA scores > −10 and < 10), with no overall synergy (HSA scores > 10) or antagonism (HSA scores < −10) observed (results from all models and replicates combined). Different compounds were the most effective with cisplatin depending on the cell line tested; ganetespib in PH003 cells (HSA score = 2.8), fimepinostat in PH142 cells (HSA score = 3.0), SRA737 in PH419 cells (HSA = 5.3), and adavosertib in #105 cells (HSA = 3.1) (Supplementary Figure S6a and Supplementary Table S8). Combined sensitivity scores (CSS) were also generated, which give a robust measure of drug combination efficacy (up to a maximum value of 100) for each combination [44]. Cisplatin and ganetespib was the most effective combination for PH142 and #105 cells (CSS = 63.2 and 97.0, respectively), cisplatin and adavosertib was the best combination for PH419 cells (CSS = 53.4), and cisplatin and fimepinostat was the best combination for PH003 cells (CSS = 86.4) (Supplementary Figure S6b and Supplementary Table S9). Synergy-sensitivity plots (SS plots) were also visualised to identify the most potent combinations, taking both HSA and CSS values into account. These plots identified ganetespib as the strongest combination with cisplatin in OCS cell lines (Supplementary Figure S6c).

In the initial drug screens, the most effective eribulin-based combinations involved inhibitors of EGFR, CDKs, MEK, EZH2, and calcium channels (Fig. 1c, Supplementary Table S6). Therefore, we selected the following six drugs for validation with eribulin in our human OCS cell lines: pimozide and manidipine (calcium channel blockers), tazemetostat (EZH2i), ryuvidine (CDK4/SETD8/KMT5Ai), erlotinib (EGFRi) and mirdametinib (MEKi). Further down the list in the validation screen were a number of HDAC inhibitors (HDACi) as well as a FAK inhibitor (FAKi) (Supplementary Table S6), so we also included panobinostat (HDACi) and PF-562,271 (FAKi). Eribulin-based combinations were tested in three OCS cell lines (PH003, PH419 and #105), as well as the two HGSOC cell lines (OVCAR8 and PEO4) for comparison. Eribulin has activity in HGSOC as well as OCS preclinical models [11, 30], and as we and others have shown previously, eribulin can also reverse EMT [11, 26]. We therefore hypothesised that eribulin-based combinations would be more effective in OCS than HGSOC. In contrast to cisplatin-based combinations, eribulin-based combinations involving erlotinib or mirdametinib were the most effective in most cell lines (PH003, OVCAR8 and PEO4). Indeed, these two combinations were synergistic in PH003 (HSA scores = 10.7 and 11.2, respectively) and OVCAR8 cells (HSA scores = 15.7 and 11.6, respectively), and additive in PEO4 cells (HSA scores = 7.9 and 8.2, respectively). In the PH419 cell line, panobinostat and mirdametinib were the most effective combinations with eribulin, demonstrating mild synergy (HSA scores = 11.2 and 10.2, respectively). In the highly resistant #105 cell line, ryuvidine demonstrated strong synergy with eribulin (HSA score = 15.8), and the combination of eribulin with panobinostat was additive (HSA score = 8.7) (Supplementary Figure S6d and Supplementary Table S8). CSS indicated that eribulin and pimozide was most effective in #105, PH419, OVCAR8 and PEO4 cells (CSS = 73.3, 74.7, 90.7 and 74.8, respectively) and eribulin and panobinostat was most effective in PH003 cells (CSS = 86.1) (Supplementary Figure S6e and Supplementary Table S9). However, the SS plot indicated that eribulin plus panobinostat was the most effective combination overall (Supplementary Figure S6f). Despite the EMT-reversing capability of eribulin, the OCS cell lines were not more sensitive than the HGSOC cell lines to the eribulin-based combinations tested.

### Combination drug screening in OCS and HGSOC organoid models indicated eribulin-based combinations were more effective in OCS

**Fig. 4:**
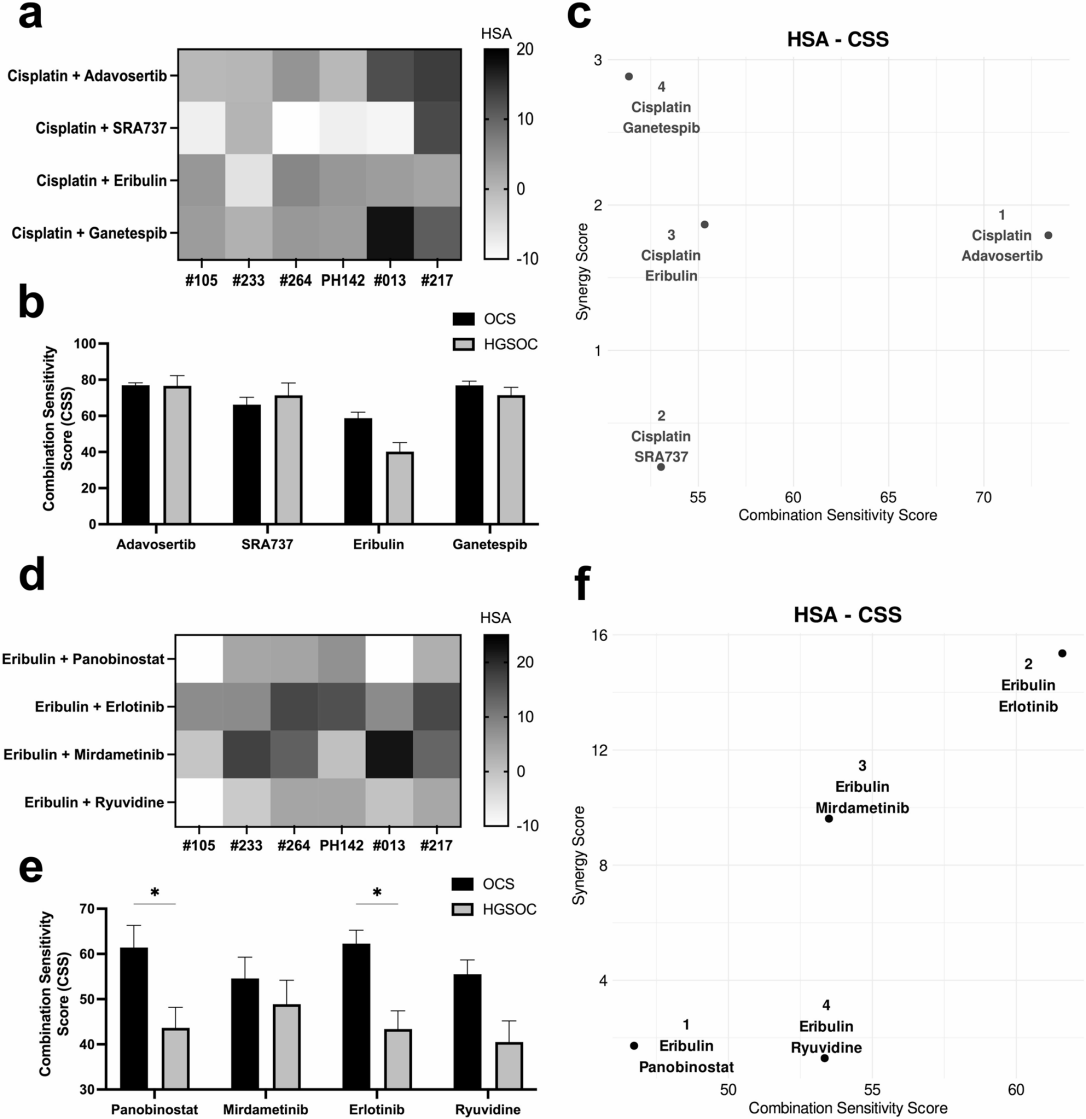
Validation of drugs that potentiate cisplatin and eribulin response in organoid models. OCS and HGSOC organoids were treated with 6-point dose range of cisplatin or eribulin in combination with 6-point dose range of selected compounds and HSA and CSS values were calculated; (**a**) HSA synergy heatmap for cisplatin-based combinations; (**b**) Bar graph comparing drug CSS values for OCS versus HGSOC organoids tested with cisplatin-based combinations; (**c**) SS plot for cisplatin-based combinations in OCS organoid models; (**d**) HSA synergy heatmap for eribulin-based combinations; (**e**) Bar graph comparing drug CSS values for OCS versus HGSOC organoids tested with eribulin-based combinations; (**f**) SS plot for eribulin-based combinations in OCS organoid models. *p < 0.05. OCS, ovarian carcinosarcoma; HGSOC, high-grade serous ovarian cancer; HSA, highest single agent; CSS, combination sensitivity score; SS, synergy score

As mentioned previously, organoids provide a more accurate prediction of therapeutic responses [40]. Therefore, we selected four OCS organoid models (PH142, #105, #233 and #264) to validate the four most effective cisplatin-based combinations identified in the cell lines. We included two HGSOC organoid models (#013 and #217) for comparison. The cisplatin-based combinations involved adavosertib, SRA737, ganetespib and eribulin. Where possible, we also included imaging of the organoids in order to determine the accuracy of our endpoint CTG data, which also allowed us to track individual organoids and exclude outliers (Supplementary Figure S7). Adavosertib and ganetespib demonstrated synergy with cisplatin in the HGSOC models (HSA scores = 14.7 and 11.0, respectively). SRA737 and adavosertib were the most effective cisplatin-based combinations in the OCS models, however, responses were only additive (HSA scores = 2.4 and 1.9, respectively) (Fig. 4a and Supplementary Table S10). Bliss and ZIP scores are also shown in Supplementary Table S10. CSS analysis ranked SRA737 and adavosertib as the most effective cisplatin-based combinations in HGSOC organoid models (CSS = 90.5 and 85.6, respectively) and adavosertib and eribulin were the most effective cisplatin-based combinations in OCS models (CSS = 73.4 and 55.3, respectively; Fig. 4b and Supplementary Table S11). The SS plot indicated that cisplatin plus adavosertib was the most effective combination for OCS overall, however, there was little difference between the scores for adavosertib, ganetespib and eribulin, with these 3 combinations ranking closely together for synergy and sensitivity (Fig. 4c).

Finally, we selected four rational eribulin-based combinations to validate in our OCS organoid models, with two HGSOC organoid models again being included for comparison. The eribulin-based combinations involved panobinostat, erlotinib, mirdametinib and ryuvidine. Mirdametinib and erlotinib demonstrated synergy with eribulin in the HGSOC models (HSA scores = 19.1 and 14.2, respectively) as well as the OCS models (HSA scores = 11.6 and 19.5, respectively) (Fig. 4d and Supplementary Table S10). CSS analysis also ranked erlotinib and mirdametinib as the most effective eribulin-based combinations in both HGSOC models (CSS = 51.2 and 51.0, respectively) and OCS organoid models (CSS = 57.6 and 47.2, respectively) (Fig. 4e and Supplementary Table S11). Comparing responses between HGSOC and OCS models indicated that erlotinib or panobinostat combined with eribulin were more effective in OCS than HGSOC (*p* = 0.032 and 0.0194, respectively; Fig. 4e). Furthermore, all eribulin-based combinations combined were significantly more effective in OCS models compared to HGSOC models (*p* < 0.0001). The SS plot indicated that eribulin plus erlotinib was the most effective combination in OCS overall, closely followed by eribulin and mirdametinib (Fig. 4f).

### Cisplatin- and eribulin-based combination therapies induced genomic instability in OCS cells

**Fig. 5:**
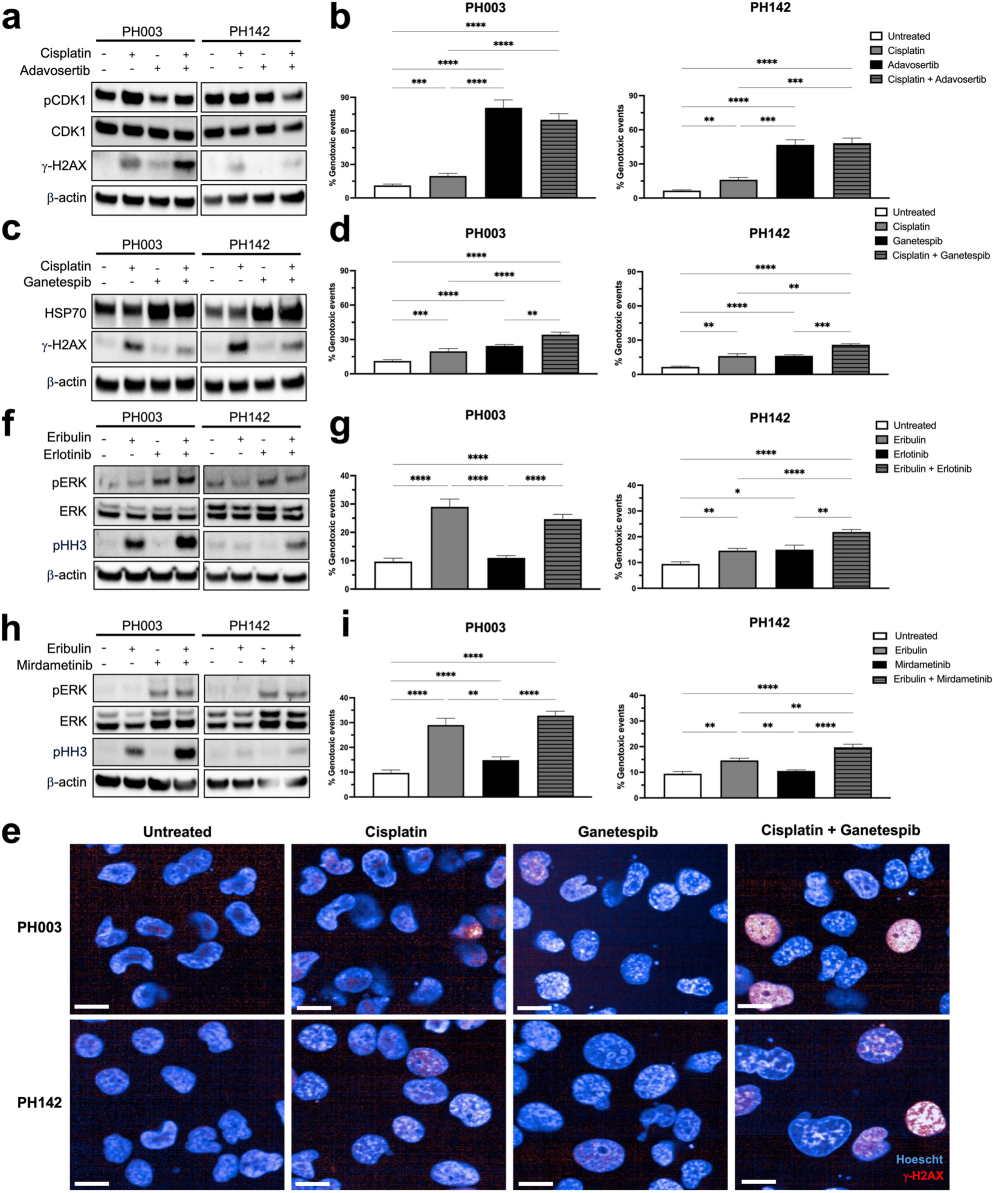
Assessment of drug on target and genomic instability following cisplatin- and eribulin-based combinations. PH003 and PH142 OCS cells were treated with EC50 doses of each drug and lysates collected or cells stained by IF after 24 h; (**a**) Expression of pCDK1, CDK1 and γ-H2AX following treatment with cisplatin and adavosertib was determined by Western Blot analysis. β-actin was used as a loading control; (**b**) Quantification of genomic instability following cisplatin and adavosertib treatment; (**c**) Expression of HSP70 and γ-H2AX following treatment with cisplatin and ganetespib was determined by Western Blot analysis. β-actin was used as a loading control; (**d**) Quantification of genomic instability following cisplatin and ganetespib treatment; (**e**) Representative images of γ-H2AX (red) and Hoechst (blue) staining in OCS cells untreated and treated with cisplatin, ganetespib and the combination; (**f**) Expression of pERK, ERK and pHH3 following treatment with eribulin and erlotinib was determined by Western Blot analysis. β-actin was used as a loading control; (**g**) Quantification of genomic instability following eribulin and erlotinib treatment; (**h**) Expression of pERK, ERK and pHH3 following treatment with eribulin and mirdametinib was determined by Western Blot analysis. β-actin was used as a loading control; (**i**) Quantification of genomic instability following eribulin and mirdametinib treatment. For IF, total number of events (micronuclei, 4 N nuclei and pan-H2AX staining) were calculated in *n* = 12; 4 independent wells, from 3 independent experiments. Data is mean + SEM. IF, immunofluorescence. *p < 0.05, **p < 0.01, ***p < 0.001, ****p < 0.0001. Scale bar = 10 μm

For the most effective combination therapies, drug on-target activity and DNA damage were assessed by Western blotting with relevant antibodies, and features of genomic instability were assessed by immunofluorescent staining with γ-H2Ax in PH003 and PH142 OCS cells. For the combination of cisplatin and adavosertib, we used γ-H2Ax and phospho-CDK1 (pCDK1) expression as indicators of cisplatin and adavosertib activity, respectively [45, 46]. Cisplatin treatment induced γ-H2Ax expression in both cell lines as expected, with increased γ-H2Ax expression evident with adavosertib combination treatment in PH003 cells (Fig. 5a). Adavosertib treatment resulted in reduced pCDK1 expression in PH003 cells only, however, in combination with cisplatin, pCDK1 levels were reduced compared to cisplatin treatment alone in both cell lines, indicating that the presence of adavosertib reduced cisplatin-mediated G2/M arrest (Fig. 5a). Cisplatin and adavosertib combination treatment resulted in a significant increase in genomic instability (quantified as the total percentage of abnormal cellular features, such as micronuclei formation, γ-H2Ax foci and pan-H2AX induction, and the presence of enlarged (≥ 4 N) nuclei) compared to cisplatin treatment alone, however, most of the damage was due to adavosertib treatment (Fig. 5b).

For the combination of cisplatin and ganetespib, we used HSP70 as a surrogate marker for HSP90 inhibition [47], with increased HSP70 expression observed whenever ganetespib was present (Fig. 5c). Cisplatin treatment again induced γ-H2Ax expression in both cell lines as expected, however, no increases in γ-H2Ax expression were observed following combination treatment (Fig. 5c). Despite this, cisplatin and ganetespib combination treatment resulted in a significant increase in genomic instability compared to cisplatin and ganetespib single agent treatment (Fig. 5d + e).

For the combination of eribulin and erlotinib, we used phospho-histone H3 (pHH3) and phospho-ERK (pERK) expression as indicators of eribulin and erlotinib activity, respectively [48–50]. Eribulin treatment resulted in a dramatic increase in pHH3 expression in PH003 cells, indicating effective mitotic arrest due to the microtubule targeting action of eribulin (Fig. 5f). Increased pHH3 expression was not observed in PH142 cells following eribulin treatment alone but was increased following combination treatment with erlotinib (Fig. 5f). Somewhat unexpectedly, pERK expression was increased following erlotinib treatment, both single agent and in combination with eribulin (Fig. 5f). Nevertheless, eribulin and erlotinib combination treatment resulted in a significant increase in genomic instability compared to erlotinib alone in PH003 cells and compared to both erlotinib and eribulin as single agents in PH142 cells (Fig. 5g).

For the combination of eribulin and the MEKi mirdametinib, we again used pHH3 and pERK expression as indicators of eribulin and mirdametinib activity, respectively [50, 51]. Eribulin treatment resulted in increased pHH3 expression in PH003 and PH142 cells, with further increased pHH3 expression observed in PH003 cells following eribulin and mirdametinib combination treatment (Fig. 5h). Unexpectedly, as we saw with erlotinib treatment, pERK expression was increased following mirdametinib treatment, both single agent and in combination with eribulin (Fig. 5h). Nevertheless, eribulin and mirdametinib combination treatment resulted in a significant increase in genomic instability compared to mirdametinib alone in PH003 cells and compared to both mirdametinib and eribulin as single agents in PH142 cells (Fig. 5i).

### The combination of eribulin and erlotinib demonstrated modest improvements in survival in OCS PDX models without RAS mutations

**Fig. 6:**
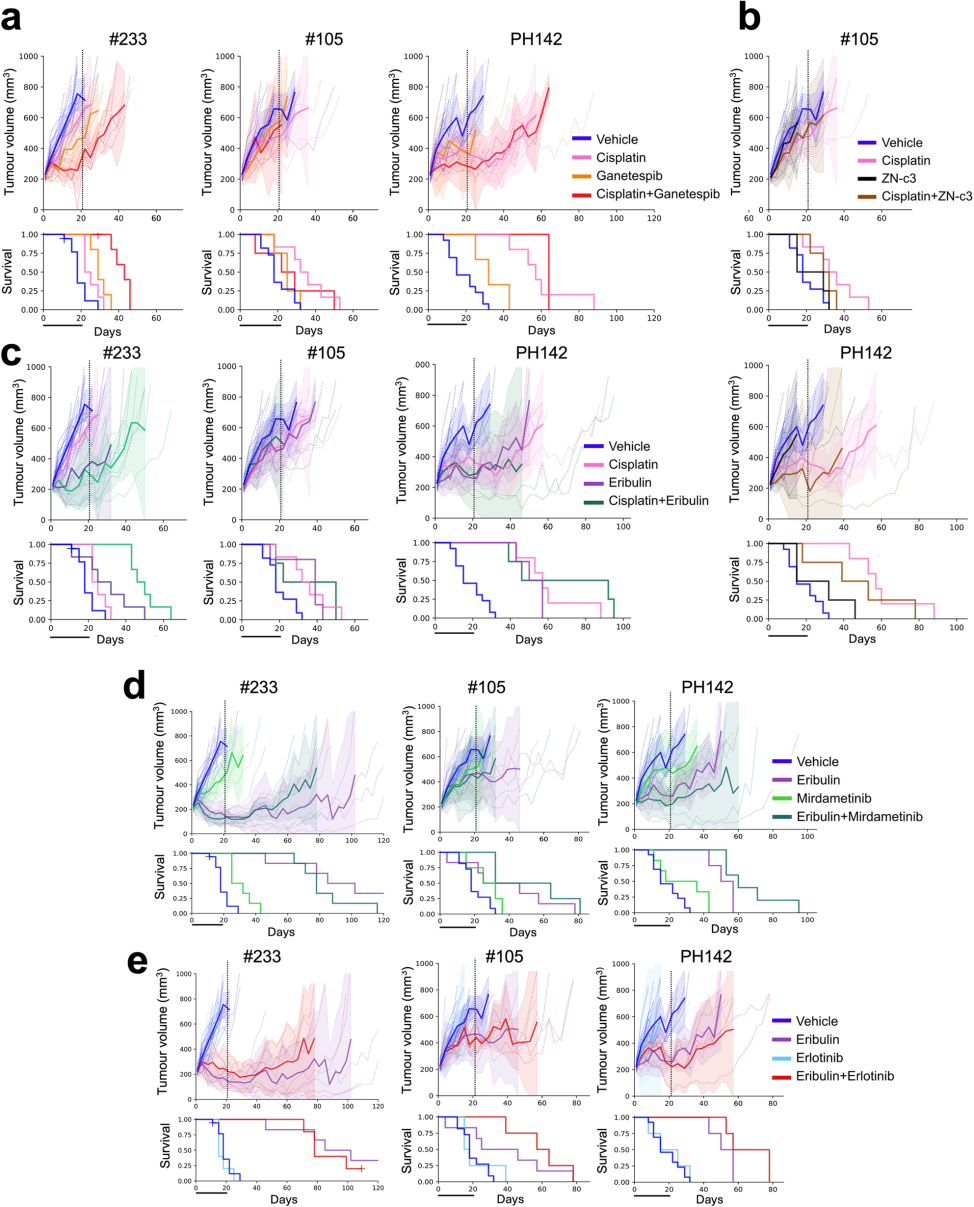
In vivo validation of cisplatin- and eribulin-based combinations. In vivo treatment of OCS PDX models with (**a**) cisplatin and ganetespib, including vehicle and single agent treatment arms; (**b**) cisplatin and ZN-c3 including vehicle and single agent treatment arms; (**c**) cisplatin and eribulin, including vehicle and single agent treatment arms. (**d**) eribulin and mirdametinib, including vehicle and single agent treatment arms; (**e**) eribulin and erlotinib, including vehicle and single agent treatment arms. Top graphs display tumour growth over time from initiation of treatment and Kaplan-Meier survival curves are shown below. *p < 0.05, **p < 0.01

We used OCS PDX models PH142, #105 and #233 for validation of combination treatments in vivo, as these have a range of responses to single-agent cisplatin and eribulin. The combination of cisplatin and ganetespib resulted in a significant improvement in TTH and survival compared to vehicle and single agent treatment in model #233 (TTH = 18 days for vehicle treatment, 25 days for cisplatin treatment, 29 days for ganetespib treatment, and 43 days for combination treatment; *p* = 0.0005 and 0.0029 for the combination vs. cisplatin and ganetespib single agents, respectively), however, no improved survival was observed in the PH142 or #105 models (Fig. 6a; Table 3). We subsequently identified #105 as having high expression of *ABCB1* [33], a gene that is known to be responsible for drug resistance [52].

**Table 3:** Combination treatment statistics for cisplatin. Statistics for the PDX treatment data presented in Fig. 6. TTH, time to harvest (days). *P* values for Kaplan-Meier survival curves were determined using the Log-Rank test. *P* values in bold indicate a significant difference (*P* value < 0.05)

PDX model	Treatment	*n*	Median TTH	*p* value vs. vehicle	*p* value vs. cisplatin	*p* value vs. ganetespib	*p* value vs. eribulin	*p* value vs. ZN-c3
#233	Vehicle	18	18					
	Cisplatin	6	25	**0.026**				
	Ganetespib	5	29	**0.0025**	0.13			
	Eribulin	6	32	**0.014**	0.25			
	Cisplatin + Ganetespib	6	43	**0.00005**	**0.0005**	**0.0029**		
	Cisplatin + Eribulin	6	50	**0.00005**	**0.0004**		**0.021**	
#105	Vehicle	10	18					
	Cisplatin	6	36	**0.0069**				
	Ganetespib	4	25	0.33	0.086			
	Eribulin	5	39	**0.0065**	0.98			
	ZN-c3	4	15	0.57	0.07			
	Cisplatin + Ganetespib	4	22	0.29	0.45	0.75		
	Cisplatin + Eribulin	4	22	0.06	0.98		0.37	
	Cisplatin + ZN-c3	4	29	**0.048**	0.31			0.28
PH142	Vehicle	13	15					
	Cisplatin	5	57	**0.0003**				
	Ganetespib	3	32	**0.039**	**0.014**			
	Eribulin	4	50	**0.001**	0.31			
	ZN-c3	4	15	**0.12**	**0.011**			
	Cisplatin + Ganetespib	3	64	**0.0034**	0.28	**0.025**		
	Cisplatin + Eribulin	4	46	**0.001**	0.37		0.47	
	Cisplatin + ZN-c3	4	39	**0.011**	0.38			0.14

For in vivo assessment of response to cisplatin plus WEE1i, we decided to test the WEE1i ZN-c3 (azenosertib), rather than adavosertib. This was due to ZN-c3 having higher selectivity for WEE1 and likely a better safety profile [53], and importantly, demonstrating impressive response rates and duration of response in ovarian cancer (NCT05128825; clinicaltrials.gov). The combination of cisplatin and ZN-c3 did not result in increased survival as compared to cisplatin or ZN-c3 alone in either #105 (TTH = 18 days for vehicle treatment, 36 days for cisplatin treatment, 15 days for ZN-c3 treatment, 29 days for combination treatment; *p* = 0.31 and 0.28 for the combination vs. cisplatin and ZN-c3 single agents, respectively) or PH142 (TTH = 15 days for vehicle treatment, 57 days for cisplatin treatment, 15 days for ZN-c3 treatment, 39 days for combination treatment; *p* = 0.38 and 0.14 for the combination vs. cisplatin and ZN-c3 single agents, respectively) (Fig. 6b; Table 3).

Lastly, the combination of cisplatin and eribulin resulted in a significant improvement in survival compared to vehicle and single agent treatments in model #233 (TTH = 18 days for vehicle treatment, 25 days for cisplatin treatment, 32 days for eribulin treatment, and 50 days for combination treatment; *p* = 0.0004 and 0.021 for the combination vs. cisplatin and eribulin single agents, respectively; Fig. 6c; Table 3).

Disappointingly, the combination of eribulin and mirdametinib had no significant effect on TTH or survival in any model tested (although TTH for the combination was significantly longer compared to mirdametinib single agent for #233 and PH142; *p* = 0.0004 and 0.0019, respectively), it was not significantly different compared to eribulin single agent (*p* = 0.22 and 0.096, respectively; Fig. 6d; Table 4). However, mixed responses to this combination were observed in PDX PH142 potentially reflecting heterogeneity in this model.

**Table 4:** Combination treatment statistics for eribulin. Statistics for the PDX treatment data presented in Fig. 7. TTH, time to harvest (days). *P *values for Kaplan-Meier survival curves were determined using the Log-Rank test. *P* values in bold indicate a significant difference (*P* value < 0.05)

PDX model	Treatment	*n*	Median TTH	*p* value vs. vehicle	*p* value vs. eribulin	*p* value vs. PD0325901	*p* value vs. erlotinib
#233	Vehicle	18	18				
	Eribulin	6	102	**0.00005**			
	Mirdametinib	6	32	**0.0014**	**0.0004**		
	Erlotinib	5	15	0.31	**0.0007**		
	Eribulin + Mirdametinib	6	78	**0.00005**	0.22	**0.0004**	
	Eribulin + Erlotinib	5	78	**0.0002**	0.58		**0.0015**
#105	Vehicle	11	18				
	Eribulin	6	46	**0.050**			
	Mirdametinib	4	25	0.14	0.31		
	Erlotinib	4	15	0.64	0.12		
	Eribulin + Mirdametinib	4	32	**0.0038**	0.28	0.10	
	Eribulin + Erlotinib	4	57	**0.0012**	0.34		**0.016**
PH142	Vehicle	13	15				
	Eribulin	4	50	**0.001**			
	Mirdametinib	6	36	0.06	**0.013**		
	Erlotinib	4	15	0.62	**0.0067**		
	Eribulin + Mirdametinib	5	60	**0.0003**	0.096	**0.0019**	
	Eribulin + Erlotinib	4	57	**0.0010**	0.14		**0.0067**

Finally, the combination of eribulin and erlotinib resulted in modest improvements in TTH and survival compared to vehicle and single agent treatments in models PH142 and #105, although not significantly different compared to eribulin single agent (PH142, 15 days for vehicle treatment, 50 days for eribulin treatment, 15 days for erlotinib treatment, and 57 days for combination treatment; *p* = 0.14 and 0.0067 for the combination vs. eribulin and erlotinib single agents, respectively; #105, 18 days for vehicle treatment, 46 days for eribulin treatment, 15 days for erlotinib treatment, and 57 days for combination treatment; *p* = 0.34 and 0.016 for the combination vs. eribulin and erlotinib single agents, respectively (Fig. 6e; Table 4). Not surprisingly, no improvement in TTH or survival was observed for the combination of eribulin with erlotinib in model #233, as this model harbours a *KRAS* mutation (Fig. 2b; Table 2).

### Addition of a third drug increases synergy of treatment combinations in OCS cell lines and organoids, with or without KRAS aberrations

**Fig. 7:**
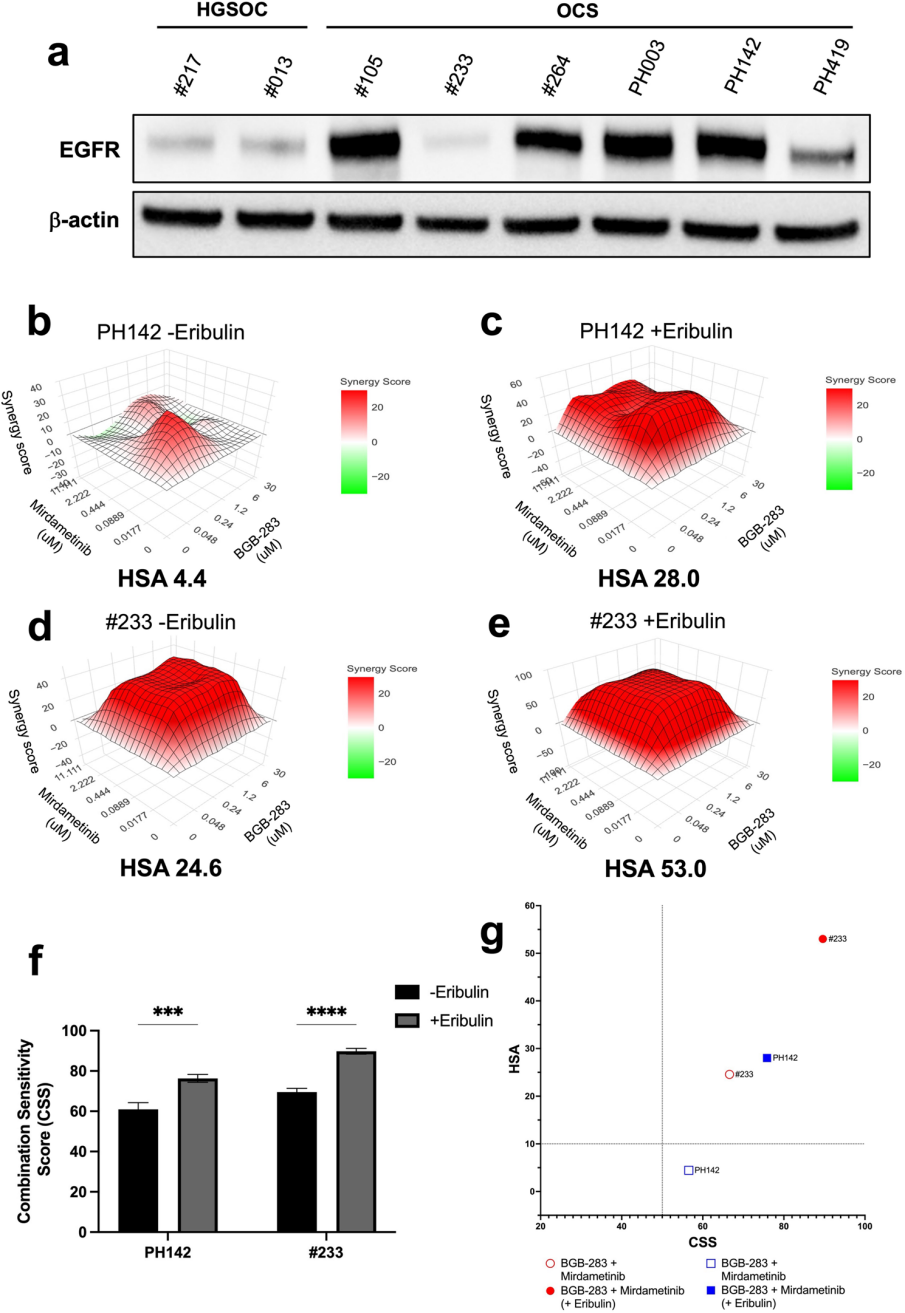
BGB-283 combined with mirdametinib overcomes KRAS mutant-mediated EGFR inhibitor resistance, and addition of eribulin further improves responses. (**a**) Western blot showing expression of EGFR in OCS and HGSOC PDX models; (**b**) HSA synergy plots for PH142 organoids treated with a BGB-283/mirdametinib drug matrix alone or (**c**) with a single dose of eribulin (0.1 nM); **d**) HSA synergy plots for #233 organoids treated with a BGB-283/mirdametinib drug matrix alone or (**e**) with a single dose of eribulin (0.1 nM); **f**) Bar graphs showing CSS values for both PH142 and #233 organoids treated with double or triple combination therapy; **g**) SS plot for PH142 and #233 organoids treated with double or triple combination therapy. HSA, highest single agent; CSS, combination sensitivity score; SS, synergy score

To further investigate this apparent reliance on EGFR and MAPK signalling (also with respect to the increased pERK expression we had observed in response to EGFR or MEK inhibition previously; Fig. 5f and h), we performed Western blotting for EGFR. All OCS models, except the KRAS mutant #233 model, had greater expression of EGFR than did the HGSOC models tested (Fig. 7a). Next, to see if we could improve responses and overcome drug resistance due to the KRAS mutation, such as in #233, we decided to investigate the combination of BGB-283 (EGFR/BRAF inhibitor) and mirdametinib, which has been a successful therapeutic strategy in KRAS mutant cancers [54]. We also decided to test this combination with a third drug, eribulin, to identify whether we could further improve responses in vitro. We chose two OCS cell lines; PH142 (KRAS wildtype) and PH003 (KRAS mutant), and two OCS organoid models; PH142 (KRAS wildtype) and #233 (KRAS mutant), to test this triple combination. These models were treated with a concentration matrix of BGB-283 and mirdametinib, with or without a single dose of eribulin (0.1 nM). In PH142 cells, the BGB-283/mirdametinib combination was moderately synergistic (HSA score = 10.6), with the addition of eribulin significantly increasing synergy (HSA score = 22.7; *p* = 0.036; Supplementary Fig. 8a + b and Supplementary Table S8). In PH003 cells, the addition of eribulin significantly increased the HSA synergy score from −1.6 (additive) to 14.5 (synergistic) (*p* = 0.0043; Supplementary Figure S8c + d). However, the addition of eribulin did not lead to any changes in the CSS for either PH142 or PH003 cells (Supplementary Figure S8e and Supplementary Table S9). The SS plot indicated that the addition of eribulin improved responses in both cell lines (Supplementary Figure S8f).

Similarly, in KRAS wildtype PH142 organoids, the combination of BGB-283/mirdametinib was additive only (HSA score = 4.4), with the addition of eribulin having a significantly synergistic effect (HSA score = 28.0; *p* = 0.0005; Fig. 7b + c). In KRAS mutant #233 organoids, the result was even more striking, with the BGB-283/mirdametinib combination demonstrating synergy (HSA score = 24.6) and the addition of eribulin significantly increasing synergy (HSA score = 53.0; *p* < 0.0001; Fig. 7d + e and Supplementary Table S10). The addition of eribulin to the combination also significantly increased CSS values for both PH142 (*p* = 0.0002) and #233 organoids (*p* < 0.0001; Fig. 7f; Supplementary Table S11). The SS plot indicated that the addition of eribulin improved responses in both organoid models (Fig. 7g). These results indicate that the triple combination of BGB-283/mirdametinib/eribulin is particularly effective in OCS models exhibiting high levels of EGFR or harbouring KRAS mutations.

## Discussion

OCS is the most aggressive gynaecological malignancy with limited treatment options. Due to its rarity, there has been a lack of preclinical models, which has hindered the development of effective treatment options. Here, we conducted drug screens on a mouse OCS cell line to identify effective single agents, as well as combinations with cisplatin (as the standard therapy for OCS is platinum-based) or eribulin (currently on trial; NCT05619913). To validate hits from our drug screens we developed a unique collection of OCS cell lines and organoid models, which to our knowledge is the largest reported collection of such models.

The initial drug screens involved 3,885 compounds each, with the top hits, as well as hits related to EMT and N-MYC (181 compounds in total), being selected for validation screens. The mouse OCS cells displayed specific sensitivities to proteasome inhibitors, antimetabolites, topoisomerase inhibitors, HSP90i and CDKi. Proteasome inhibitors (bortezomib, MG-132, ixazomib and disulfiram) and HSP90i (BIIB021) were validated as effective single agents in human OCS cell lines and organoids. In addition, inhibitors of PI3K (fimepinostat and PKI-587) and HMG-CoA reductase (fluvastatin) and a drug that targets multiple kinases simultaneously (SRC/FGFR1/PDGFRb/WEE1; PD-166285) were particularly effective in human OCS cell lines and organoids. Most models were relatively resistant to cisplatin, with the most sensitive models, unsurprisingly, being the two harbouring mutations in a *BRCA* gene, PH142 and #264 [55]. The drugs expected to target EMT, were not more effective in OCS cell lines and organoids compared to HGSOC models, suggesting alternative dominant modes of action for these drugs. On the other hand, although not significant, most drugs selected as being relevant to N-MYC were more effective in OCS organoid models compared to HGSOC organoid models. This is not surprising, as increased N-MYC expression has been associated with an induction of EMT genes [56], and we have previously shown many of our OCS models have high N-MYC expression [11]. The lack of difference observed between OCS and HGSOC cell lines may be due to the limitation of 2D cultures, where plastic has a very different structure to the natural in vivo environment, causing drastic changes in cell morphology [57].

Despite impressive responses in the OCS organoid models (in the low nanomolar range), in vivo responses to single agents were not as robust. Indeed, only one single agent, ganetespib, influenced tumour growth and mouse survival, which was significant in all three models tested, which likely warrants further investigation in the future. However, given the aggressive nature of this disease, we decided to focus on cisplatin and eribulin combinatorial treatments, as combination strategies will likely be required for effective and enduring inhibition of tumour growth.

The most effective cisplatin-based combinations for all models (OCS and HGSOC cell lines and organoids) involved adavosertib or ganetespib. These combinations were more effective in the HGSOC organoid models than the OCS models. This is somewhat expected given HGSOC is more responsive to platinum-based therapy than OCS in the clinic [4]. Many features of these tumours may explain why they are specifically sensitive to WEE1 or HSP90 inhibitors, such as *TP53* mutation (all models except #233) [58–60], *CCNE1* amplification (#201 and #105) [58, 61], *KRAS* mutation (PH003 and #233) [62, 63], *RB1* mutation (#105) [58, 64], *MYC* amplification (#201) and high N-MYC expression (OCS models [11]) [65–67]. Indeed, the combination of platinum therapy with WEE1i has already been tested for HGSOC in Phase II clinical trials, with anti-tumour efficacy observed [68–71]. Cisplatin plus ganetespib was the most effective combination in all organoid models overall and significantly increased genomic instability in OCS cells compared to single agent treatment. Despite these promising in vitro results, cisplatin plus ganetespib combination therapy was only significantly more effective than single agent treatment in 1/3 PDX models tested. We previously identified #105 as having high expression of *ABCB1* [33], the gene that encodes P-glycoprotein 1 (P-gp), also called multidrug resistance protein 1 (MDR1), a protein involved in cellular efflux of chemotherapeutic drugs [52]. Although cisplatin is not considered to be a substrate of P-gp, increased expression of P-gp has been associated with cisplatin resistance in osteosarcoma [72], and could potentially explain the lack of response observed in #105 to any cisplatin-based combinations in vivo. Previously, HSP90 inhibition has been shown to synergise with cisplatin in head and neck cancer cells, pancreatic ductal adenocarcinoma cells, gastric cancer cells, and diffuse large B-cell lymphoma cells [73–76]. In addition, the combination of platinum therapy with HSP90i has been tested in lung cancer and malignant pleural mesothelioma in Phase I trials, with some anti-tumour efficacy observed [77]. Therefore, depending on the molecular profile of the tumour, this combination could be considered as an effective therapy for OCS.

We also tested the combination of cisplatin plus eribulin in our OCS PDX models. We tested this combination based on the knowledge that OCS was less responsive than HGSOC to platinum-based chemotherapy, likely due to the presence of sarcomatous cells [4], and our previous finding that eribulin can reduce the mesenchymal characteristics of OCS cells [11]. Therefore, we hypothesised that eribulin treatment may make OCS more sensitive to platinum-based chemotherapy. Indeed, we found that eribulin plus cisplatin moderately improved survival compared to single agent treatments in two out of three PDX models, with a significant effect being observed in one out of three models.

The most effective eribulin-based combinations for all models involved erlotinib and mirdametinib, independently. These combinations were more effective in the OCS organoid models than the HGSOC models. This is somewhat expected given our previous finding that OCS have increased expression of an EMT signature and eribulin can reverse EMT in OCS preclinical models [11]. Features of these tumours that may explain why they are specifically sensitive to MEK inhibitors, include *KRAS* mutation (OVCAR8, PH003 and #233) [78, 79] and high expression of EGFR (all OCS models except #233) [80]. With respect to EGFR inhibition, most of the OCS models did have higher EGFR expression than the HGSOC models. The higher EGFR expression, and responses observed to EGFR inhibition in combination with eribulin, suggest that OCS may be particularly reliant on this pathway for their growth and survival. Eribulin plus erlotinib was the most effective combination when averaging responses observed in all organoid models. Further analysis indicated a significant increase in genomic instability following treatment of OCS cells with the combination compared to erlotinib single agent treatment (and eribulin single agent treatment in one cell line). Reliance on EGFR signalling may also explain why ganetespib was the most effective single agent, as EGFR is an HSP90 client protein [42]. Ganetespib can reduce expression of EGFR and could also sensitise cells to EGFR inhibition [81], indicating this could be a viable combination worth testing in our OCS models in the future.

Despite these promising in vitro results, eribulin plus erlotinib combination therapy was only moderately more effective than single agent treatment in two out of three PDX models tested and improved survival was not observed in any of the three models tested for the combination of eribulin plus mirdametinib. For eribulin plus erlotinib, the lack of improved effect observed in model #105 can again be explained by the high expression of *ABCB1*, as eribulin is an MDR substrate [82]. The lack of combination effect observed in model #233 can be explained by the presence of an activating *KRAS* mutation, a known resistance mechanism to EGFR inhibition [83]. Furthermore, both erlotinib and mirdametinib treatment (alone and in combination with eribulin) resulted in increased MAPK signalling in the OCS cell lines, as indicated by increased pERK expression. This is an adaptive response that has been observed previously, particularly in cells reliant on EGFR signalling (especially KRAS mutant cells such as PH003), and when analysing cells after a short exposure to erlotinib [48, 49], further supporting our hypothesis that EGFR signalling is important for OCS growth and survival. It is likely that erlotinib and mirdametinib single agent treatments are activating compensatory mechanisms and negative feedback loops in OCS cells, respectively. These resistance mechanisms to EGFRi and MEKi as single agents have been observed in multiple cancer types previously [84, 85]. Furthermore, it is likely that targeting of the EGFR/MAPK pathway would be enhanced when a functional immune system is present. Our organoid models and PDX are both deficient in functional immune components, however, other research has suggested that immune cells substantially contribute to the anti-tumour effects of EGFR inhibitors [86]. For example, EGFR inhibition in non-small cell lung cancer was shown to enhance the proliferation and activation of both CD4+ and CD8+ T-cells in the tumour microenvironment [87]. This suggests that the addition of immune cells to our models could improve responses in the future.

The combination of BGB-283 (EGFR/BRAFi) with mirdametinib (MEKi) has previously been shown to prevent activation of compensatory mechanisms and negative feedback loops in KRAS mutant tumours [88, 89]. This combination, in particular when combined with eribulin, exhibited increased synergy in both OCS cell lines and organoids, indicating that addition of just a small dose of eribulin could result in an improved response. It was noted that this combination worked well in models with KRAS aberrations, as well as in KRAS wildtype models expressing high levels of EGFR, suggesting it could potentially be used as a triple combination therapy for OCS. Importantly, improved responses were observed for lower concentrations of these drugs, when used as part of a triple combination therapy, which indicates that lower combination doses may provide efficacy at tolerable doses for patients. These findings warrant further investigation, in more models in vitro as well as in vivo, which will require careful scheduling and potentially the addition of immune cells. It is clear that OCS will require a multi-targeted drug approach to have durable responses.

While the in vivo results showed only modest additive effects of the drugs at best, the synergistic effects observed in organoid models, in particular with respect to triple combinations, provide a strong rationale for further investigation of cisplatin- and eribulin-based combinations in OCS. Further, our organoid models showed more pronounced results than those observed in our cell lines. This finding was not surprising, considering that 3D organoids are better for modelling tumours with mixed carcinomatous and sarcomatous components, as 2D cell lines undergo genetic drift over time and tend to become homogenous cultures [90]. Future studies should focus on overcoming identified resistance mechanisms and exploring additional combination partners for eribulin and/or cisplatin, as well as including more OCS models to increase the scope of our data. As OCS are genomically complex, they may require triple combination therapy. Triple combinations have shown promise over double combinations in other difficult to treat cancers, such as advanced breast cancer and multiple myeloma, especially in highly mutated cancers [91–93], and the results here support triple combination therapy for OCS, and potentially other tumour types harbouring EMT, in particular to overcome drug resistance due to genetic aberrations. Moreover, the development of predictive biomarkers to identify patients most likely to benefit from these combinations will be crucial for translating these findings into clinical practice. The differential response between OCS and HGSOC to both eribulin- and cisplatin-based combinations highlights the distinct biology of these tumour types, despite having similar molecular profiles. This observation reinforces the need for continued exploration of tailored treatment strategies for OCS in the clinic, rather than continuing to rely on data from other types of ovarian cancer (which do not display EMT characteristics). These results represent a significant step forward in our understanding of OCS and potential treatment strategies and offer hope for improved outcomes in this challenging malignancy.

### Limitations of this study

This study used only a small number of models due to the rarity of OCS. Although, to our knowledge, this is the largest collection of OCS preclinical models published to date. For some cases, we were only able to successfully generate a cell line or an organoid model (not both), which sometimes limited data comparisons. Where possible, we have matched data between cases (i.e. cell lines, organoids, and PDX), though this was not always feasible. In some instances, compounds that were most effective in our original drug screens were not validated in our human preclinical models or were substituted for another compound targeting the same molecule or pathway. This was mostly due to limited clinical efficacy, unacceptable toxicity, or better activity observed for alternative drugs (for example, the use of ganetespib and ZN-c3 in our in vivo models instead of other HSP90i or WEE1i, respectively). Given the small sample size and variability among models (e.g. the proportion of carcinoma and sarcoma in each sample, which also varies between patients), it is unlikely that a single drug combination will be effective for all OCS. The inclusion of more models in future studies will be essential to clarify treatment options for patients.

## Supplementary Information

Supplementary Material 1: Supplementary Figure S1: Drug screening of a mouse OCS cell line identifies sensitivity to independent cisplatin- and eribulin-based combinations. a) Dose response curves for eribulin and cisplatin in the mouse OCS cell line (left), validation of EC20 doses (right), and surviving fractions for chosen EC20 concentrations (bottom); b) Waterfall plot of cisplatin-based combination drug effects in the initial screen. DEffect values are calculated by subtracting the effect of the compound alone from the effect of the compound in combination with cisplatin. Most effective cisplatin-based combinations are indicated on the right as well as compounds selected for the validation screens (green); c) Waterfall plot of eribulin-based combination drug effects in the validation screen. DEffect values are calculated by subtracting the effect of the compound alone from the effect of the compound in combination with eribulin. Most effective eribulin-based combinations are indicated on the right as well as compounds selected for the validation screens (green). SF, survival fraction. Supplementary Figure S2: Validation of EMT-related single agent hits from the drug screen in cell lines. a) Dose response curves for each compound in four cell lines (two OCS and two HGSOC); b) EC50 values for each of the EMT-related single agents tested in cell lines; c) Heatmap of EC50 values for each EMT-related single agent in each cell line; d) Average EC50 values for the HGSOC cell lines combined compared to OCS cell lines combined. Supplementary Figure S3: Validation of N-MYC-related single agent hits from the drug screen in cell lines. a) Dose response curves for each compound in four OCS cell lines; b) EC50 values for each of the N-MYC-related single agents tested in cell lines; c) Heatmap of EC50 values for each N-MYC-related single agent in each cell line. Supplementary Figure S4: Validation of all single agent hits from the drug screen in organoids. a) Dose response curves for EMT-related compounds in seven organoid models (two HGSOC and five OCS); b) Dose response curves for N-MYC-related compounds in nine organoid models (three HGSOC and six OCS). Supplementary Figure S5: Western blot showing increased expression of N-MYC in OCS. Expression levels of N-MYC were validated via Western blot. Supplementary Figure S6: Validation of cisplatin- and eribulin-based combinations in OCS cell lines. Cell lines were treated with 6-point dose range of cisplatin or eribulin in combination with 6-point dose range of selected compounds and HSA and CSS values were calculated; a) HSA synergy heatmap for cisplatin-based combinations in OCS cell lines; b) CSS values for cisplatin-based combinations; c) SS plot for cisplatin-based combinations in OCS cell lines; d) HSA synergy heatmap for eribulin-based combinations in OCS and HGSOC cell lines; e) CSS values for eribulin-based combinations; f) SS plot for eribulin-based combinations in OCS and HGSOC cell lines. HSA, highest single agent; CSS, combination sensitivity score; SS, synergy score. Supplementary Figure S7: Imaging and tracking of drug treated organoids. The growth and changes to architecture of the organoids were tracked over time by imaging once a day for 7 days after drugs were added. In this example, a matrix of cisplatin and adavosertib were added to #233 organoids and their growth was tracked over time. An example of the change in organoid appearance following drugging can be seen in the panels on the right. Supplementary Figure S8: Triple combination of eribulin with BGB-283/mirdametinib increases drug synergy in OCS cell lines. a) HSA synergy plots for PH142 cells treated with a BGB-283/mirdametinib drug matrix alone or (b) with a single dose of eribulin; c) HSA synergy plot for PH003 cells treated with a BGB-283/mirdametinib drug matrix alone or (d) with a single dose of eribulin; e) Bar graphs showing CSS values for both PH142 and PH003 cells treated with double or triple combination therapy; f) SS plot for PH142 and PH003 cells treated with double or triple combination therapy. HSA, highest single agent; CSS, combination sensitivity score; SS, synergy score.

Supplementary Material 2.

## Data Availability

The datasets generated and analysed during the current study are available from the corresponding author on reasonable request.

## Abbreviations

AUC: Area under the curve
CDK: Cyclin-dependent kinase
CSS: Combined sensitivity score
EC20: Drug concentration that kills 20% of cells
EGFR: Epidermal growth factor receptor
EMT: Epithelial-to-mesenchymal transition
GEMM: Genetically-engineered mouse model
HGSOC: High grade serous ovarian carcinoma
HMGA2: High-mobility-group AT-hook protein 2
HSA: Highest single agent
HSP90: Heat shock protein 90
NGS: Next generation sequencing
OCS: Ovarian carcinosarcoma
PDX: Patient-derived xenograft
pHH3: Phospho-histone H3
SFRCP: Stafford Fox Rare Cancer Program
SS: Synergy-sensitivity
TTH: Time to harvest
WES: Whole exome sequencing
WGS: Whole genome sequencing
