# Supplementary material for: High-throughput drug screening identifies EGFR/MAPK pathway targeting sensitivities in organoid models of ovarian carcinosarcoma
Source: J Exp Clin Cancer Res. 2026 Jan 6;45:32. doi: 10.1186/s13046-025-03629-8 (PMC12870163; doi:10.1186/s13046-025-03629-8)

Supplementary Figure S1

a

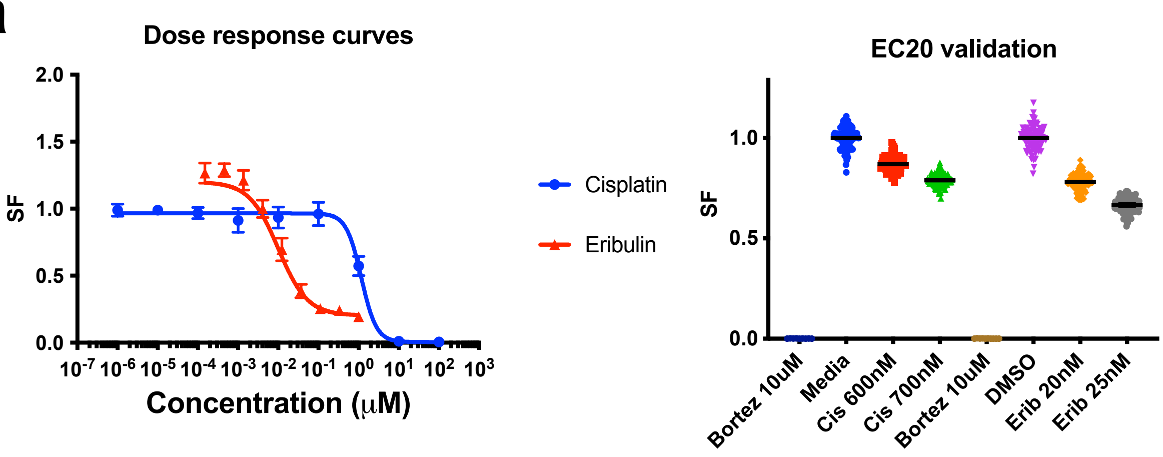

Surviving fractions (SF)

	Bortez 10uM	Media	Cis 600nM	Cis 700nM	Bortez 10uM	DMSO	Erib 20nM	Erib 25nM
Mean	6.250e-011	1.000	0.8705	0.7894	8.750e-011	1.000	0.7805	0.6665
Std. Deviation	0.001268	0.05288	0.03597	0.02976	0.001579	0.05865	0.03963	0.03566
Std. Error of Mean	0.0003170	0.005397	0.003179	0.002630	0.0003949	0.005986	0.003502	0.003152

b

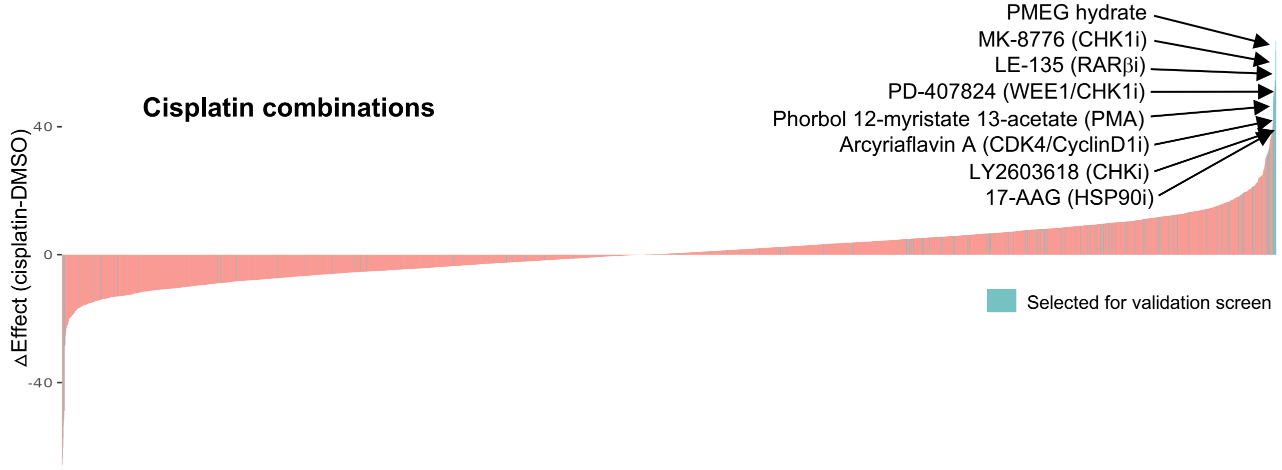

c

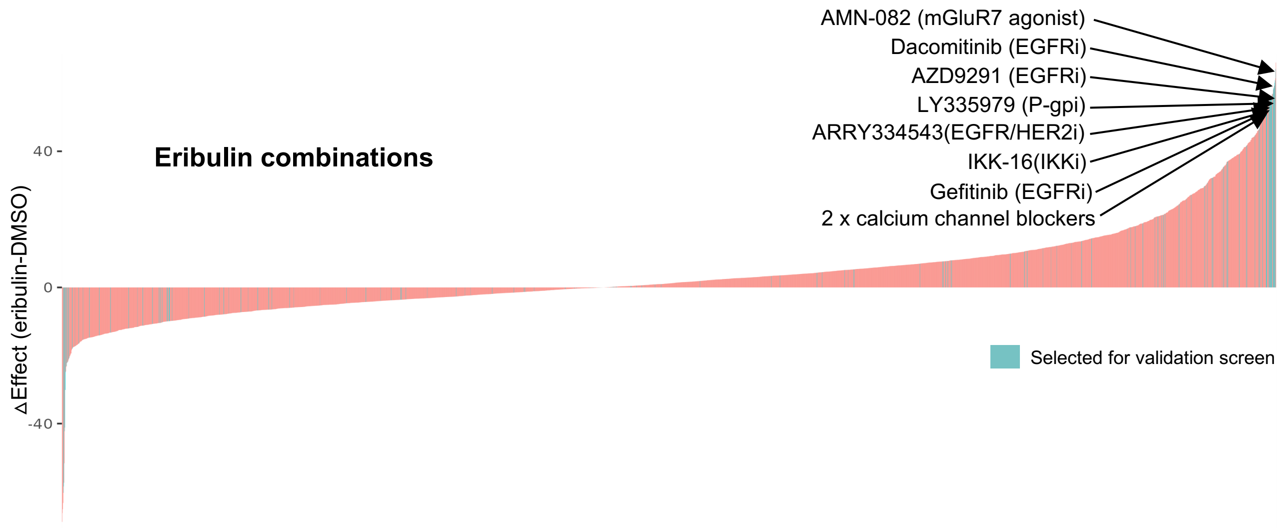

Supplementary Figure S2

a

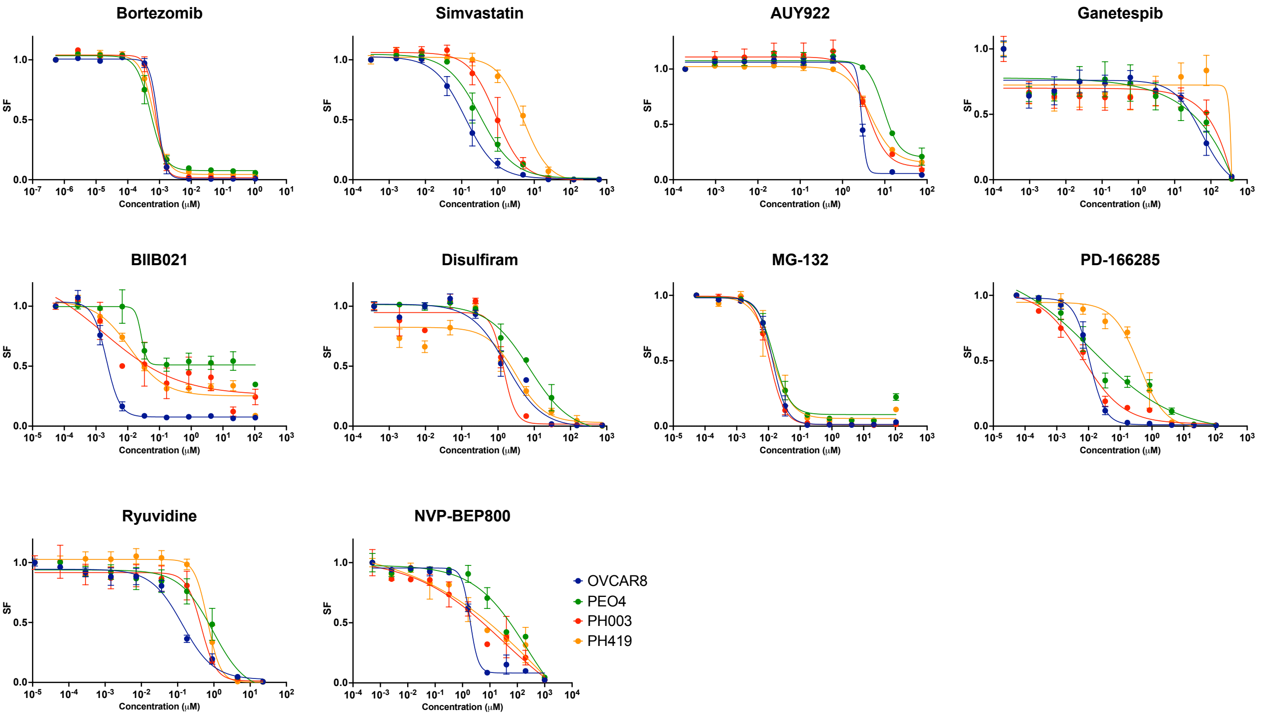

b

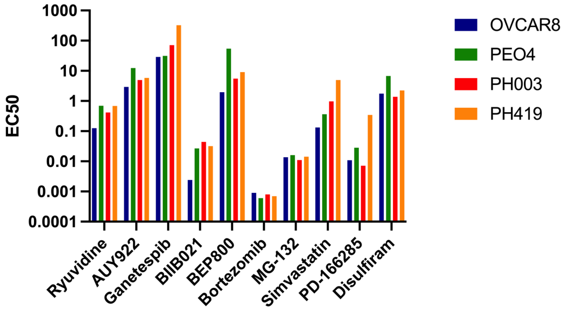

d

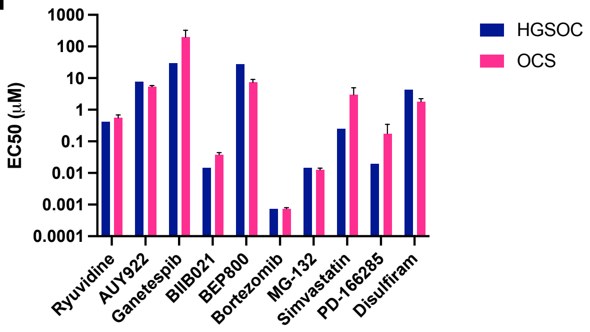

c

	Ryuvidine	AUY922	Ganetespib	BIIB021	NVP-BEP800	Bortezomib	MG-132	Simvastatin	PD-166285	Disulfiram
OVCAR8	0.1261	2.9191	28.7278	0.0024	1.9453	0.0009	0.0136	0.1333	0.0108	1.7521
PEO4	0.6993	12.3855	31.2010	0.0267	54.5705	0.0006	0.0161	0.3655	0.0284	6.7457
PH003	0.4159	4.9335	70.7671	0.0442	5.4861	0.0008	0.0110	0.9766	0.0072	1.3779
PH419	0.6830	5.8369	323.1377	0.0318	9.1167	0.0007	0.0142	4.9832	0.3458	2.2541

Supplementary Figure S3

a

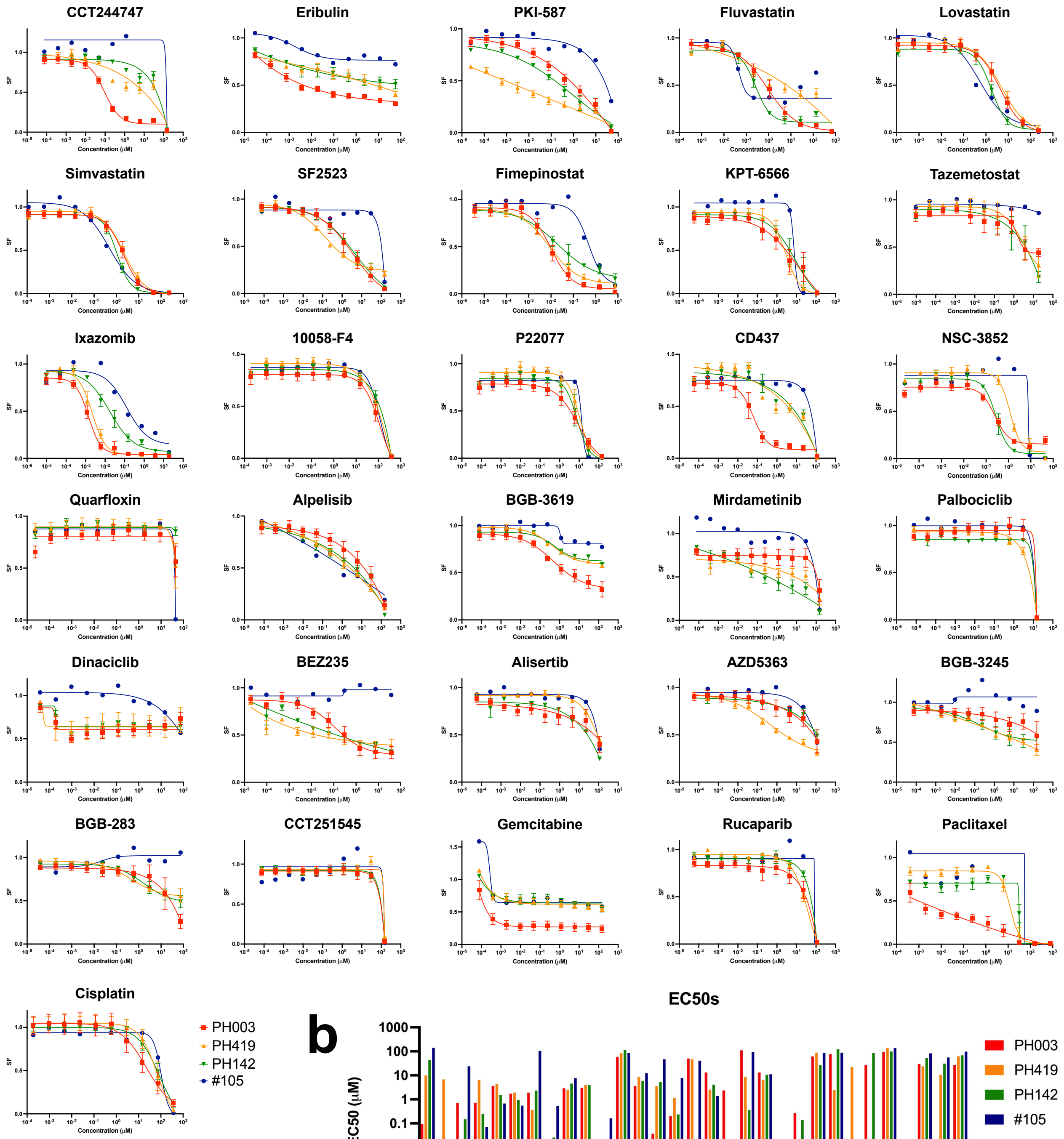

b

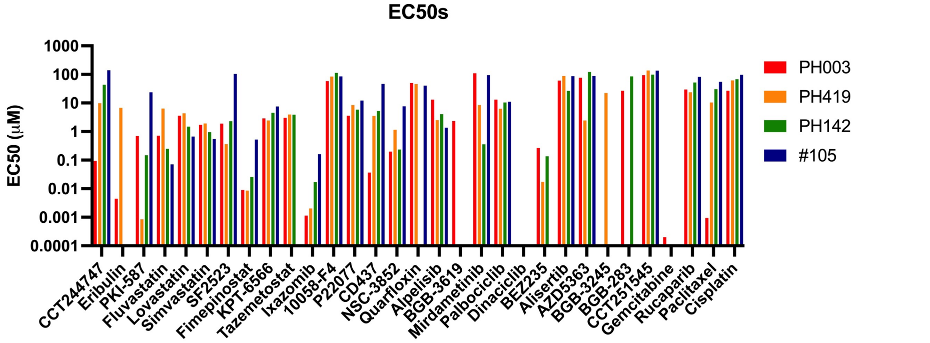

c

	CCT244747	Eribulin	PKI-587	Fluvastatin	Lovastatin	Simvastatin	SF2523	Fimepinostat	KPT-6566	Tazemetostat	Ixazomib	10058-F4	P22077	CD437	NSC-3852	Quarfloxin
PH003	0.08837847	0.001609	0.20516375	0.272832	1.329294	0.7592278	0.310415	0.003669579	0.3711897	0.427891129	0.000756592	32.4549714	1.1047132	0.004838	0.162405	40.26587
PH419	49.6716264	21.50619	0.01109833	26.38735	7.160829	2.5003191	0.809558	0.014138454	1.1269397	NR	0.001029123	90.8114429	3.2207877	7.490237	1.006431	39.20089
PH142	102.737141	0.004006	0.99027085	0.088517	0.723055	0.4625116	0.993653	0.154819896	0.852426	0.139293096	0.026096812	56.5319529	1.4067408	0.730024	0.651133	NR
#105	140.090795	NR	23.8048117	0.070715	0.670724	0.5518736	103.2945	0.525513898	7.5813635	NR	0.1616793	85.3965415	12.184774	46.5401	7.709425	40.5146

	Alpelisib	BGB-3619	Mirdametinib	Palbociclib	Dinaciclib	BEZ235	Alisertib	AZD5363	BGB-3245	BGB-283	CCT251545	Gemcitabine	Rucaparib	Paclitaxel	Cisplatin
PH003	1.26384714	0.95272	3.29423257	6.468795	NR	0.0387296	2.068434	1.53479024	9.274643	1.564342718	55.84332479	7.1922E-05	7.5945398	0.00148	7.234415
PH419	0.87805236	NR	67.8307608	6.026548	NR	39.985762	79.69355	1.237254846	NR	NR	138.0343742	NR	16.340307	6.345657	42.13209
PH142	0.72722384	NR	21.5237137	8.834028	NR	0.1137406	22.90977	30.79213341	NR	NR	73.63429107	0.0012964	40.409264	35.9641	64.65855
#105	1.36522566	NR	93.9809341	11.02003	NR	NR	86.95698	87.5570449	NR	NR	135.8500223	NR	82.381729	55.1181	96.31702

Supplementary Figure S4

a

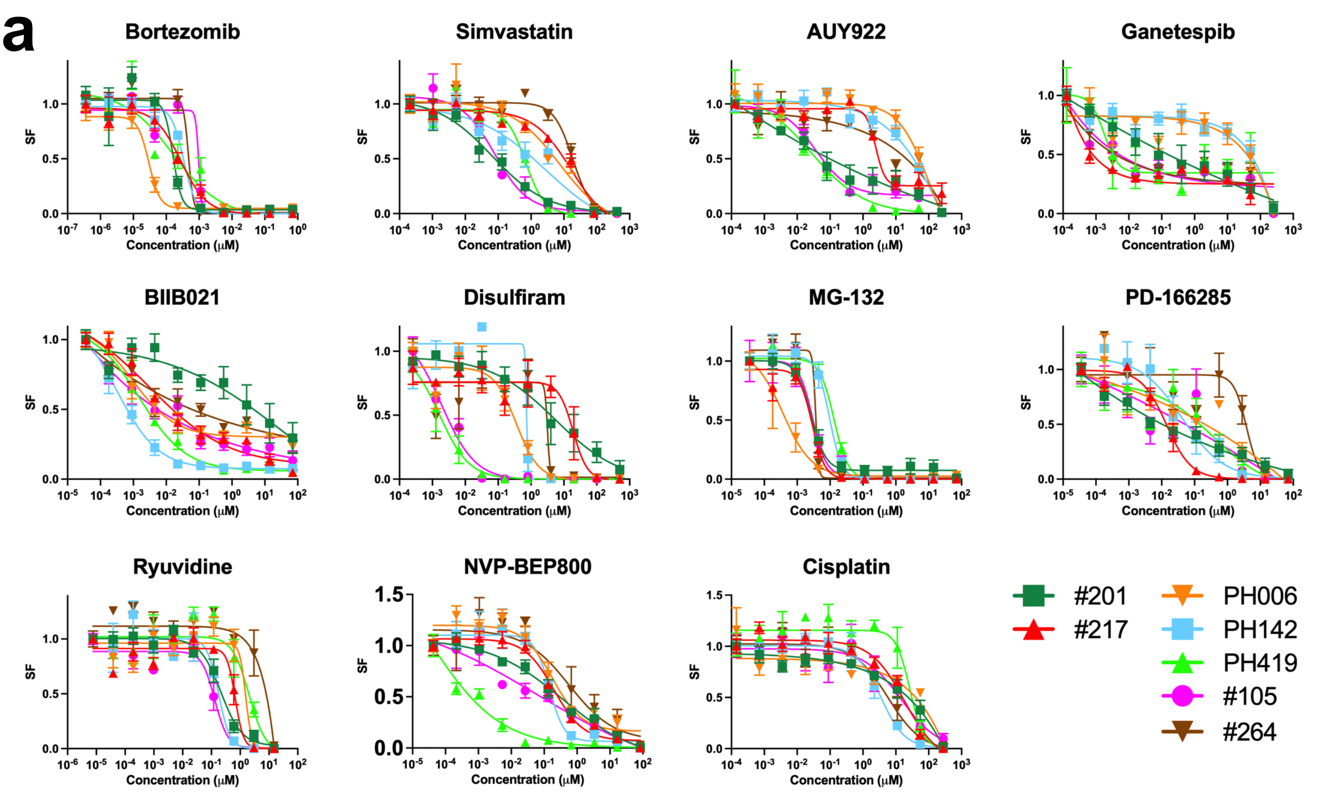

b

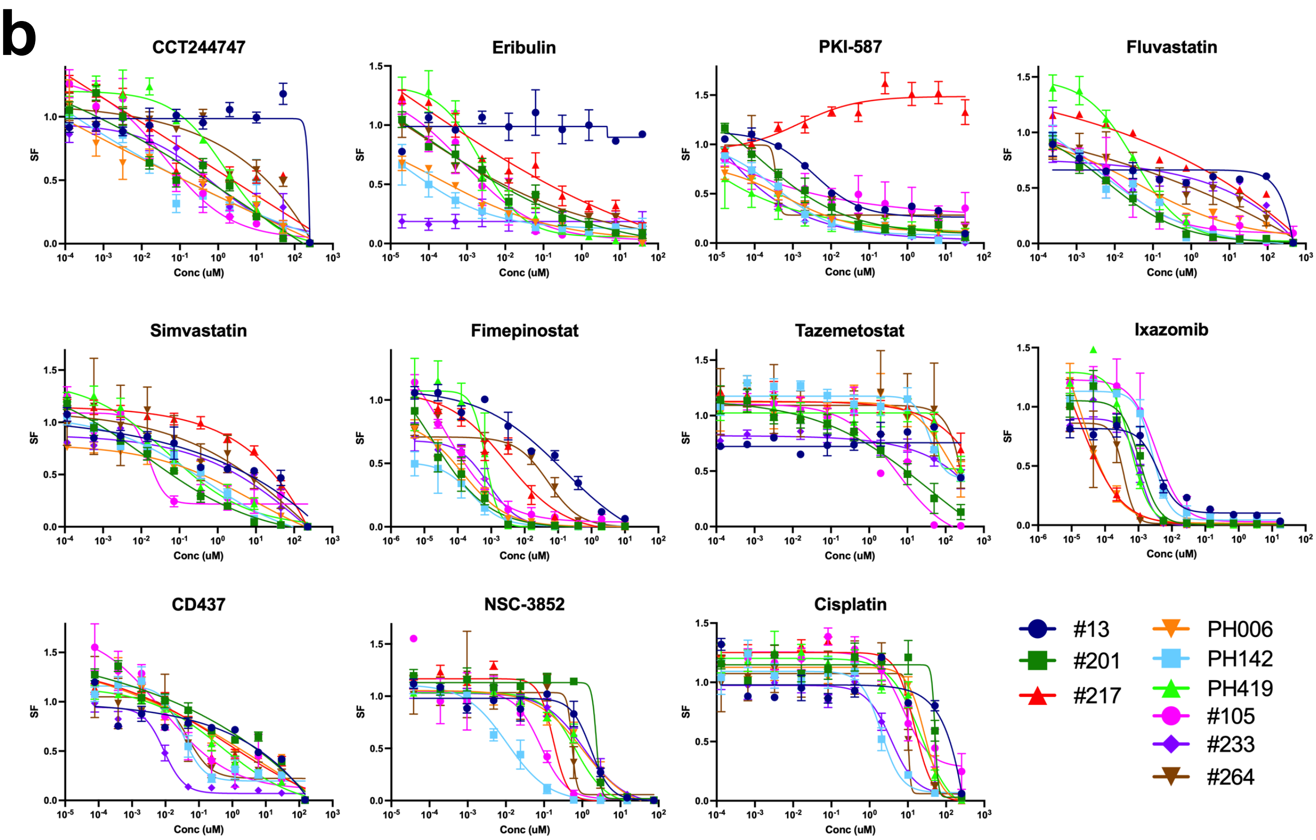

Supplementary Figure S5

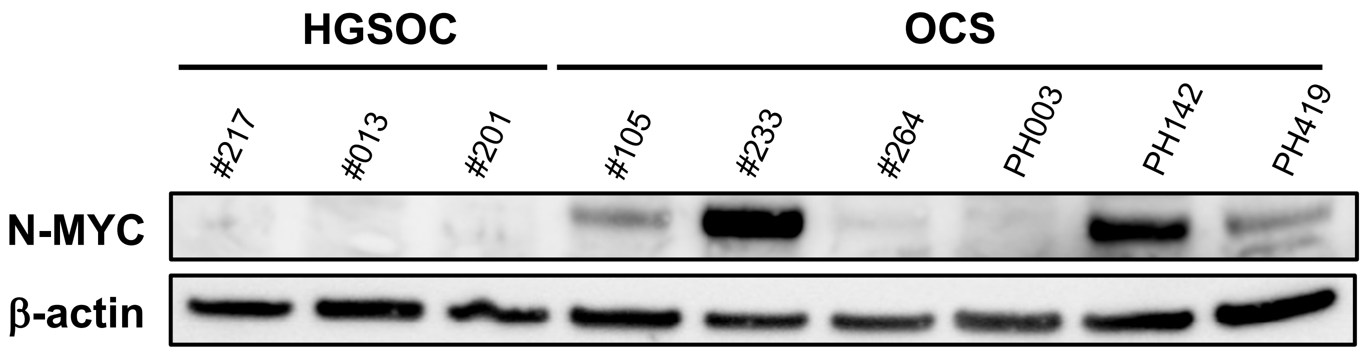

Supplementary Figure S6

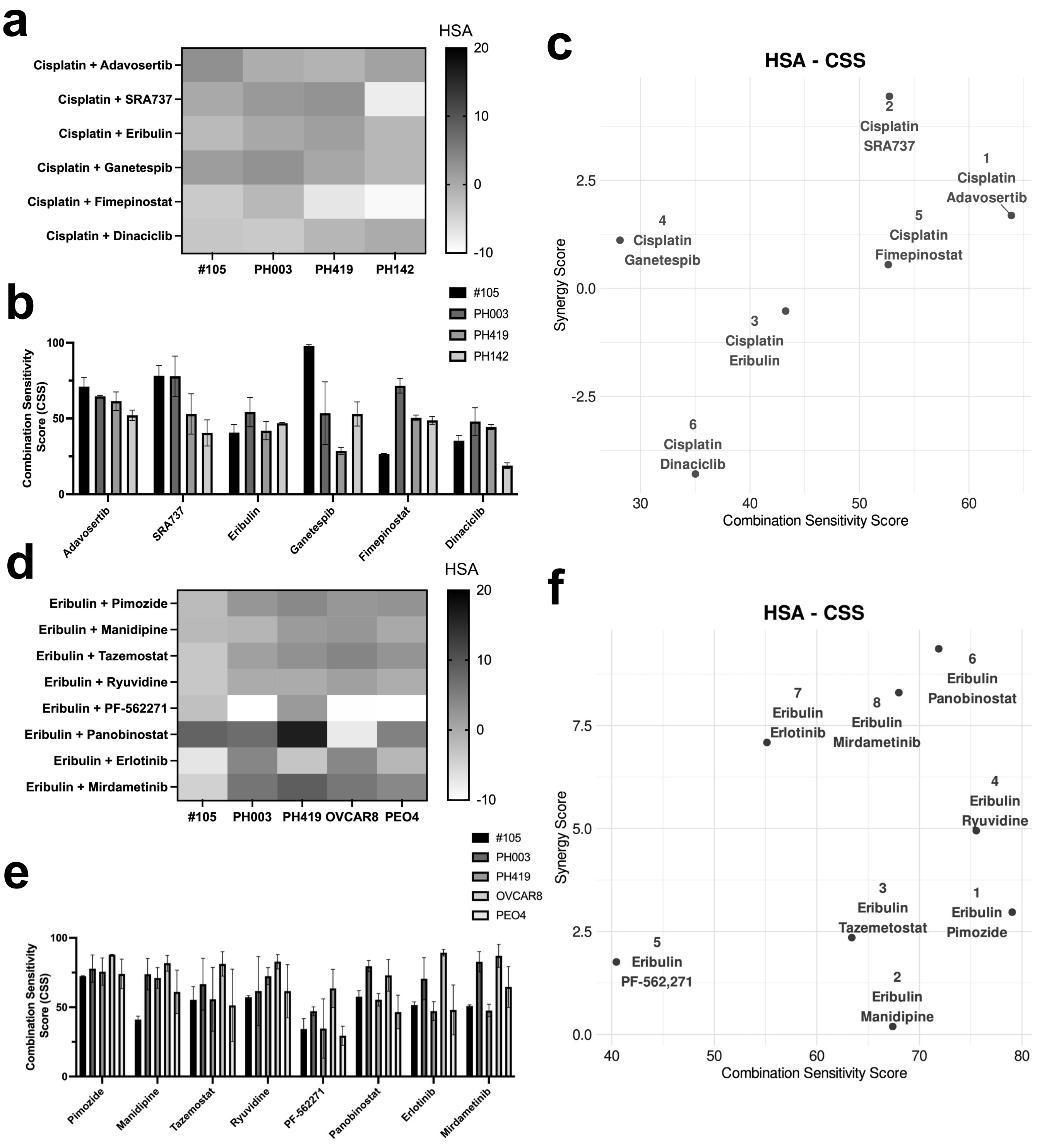

Supplementary Figure S7

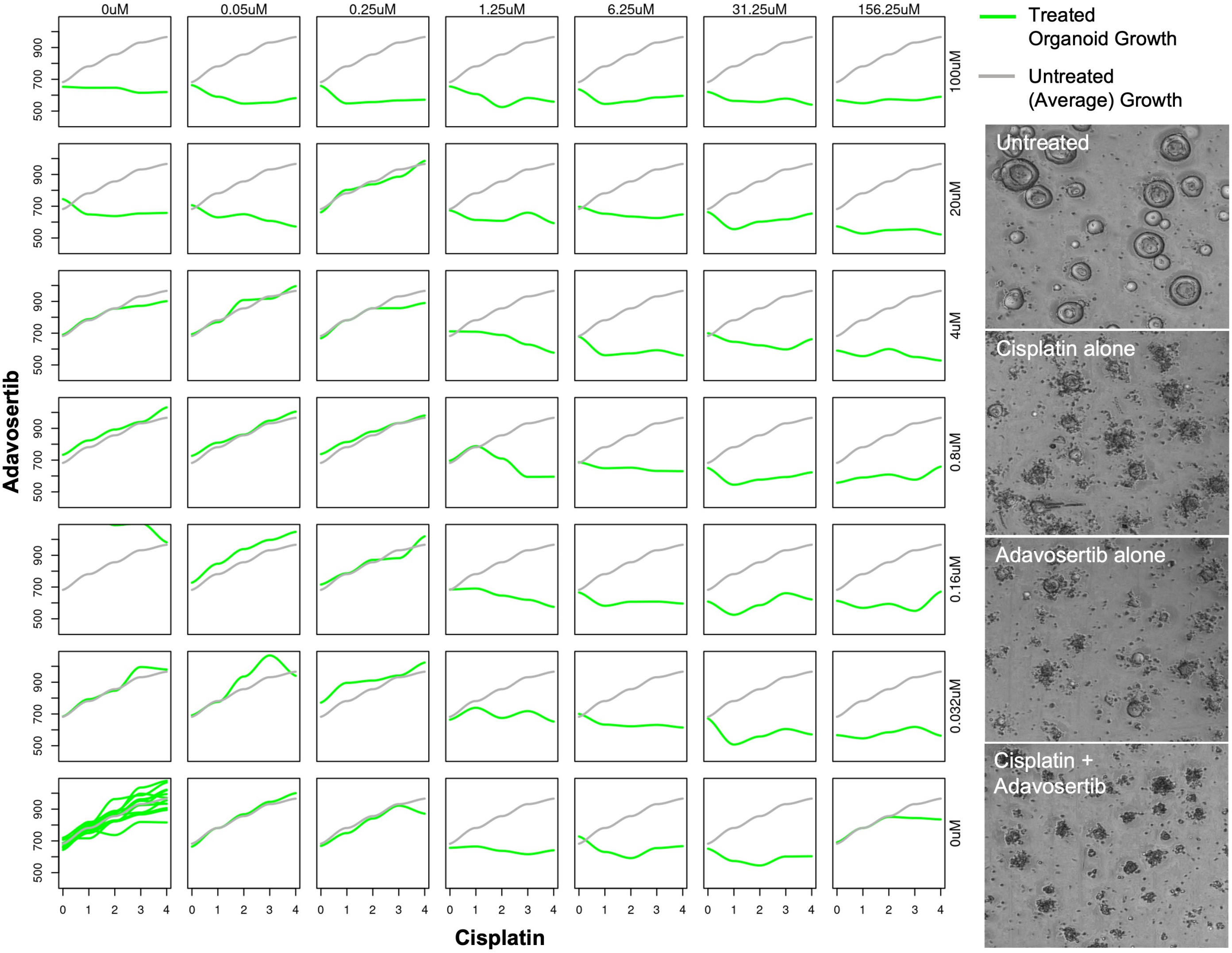

Supplementary Figure S8

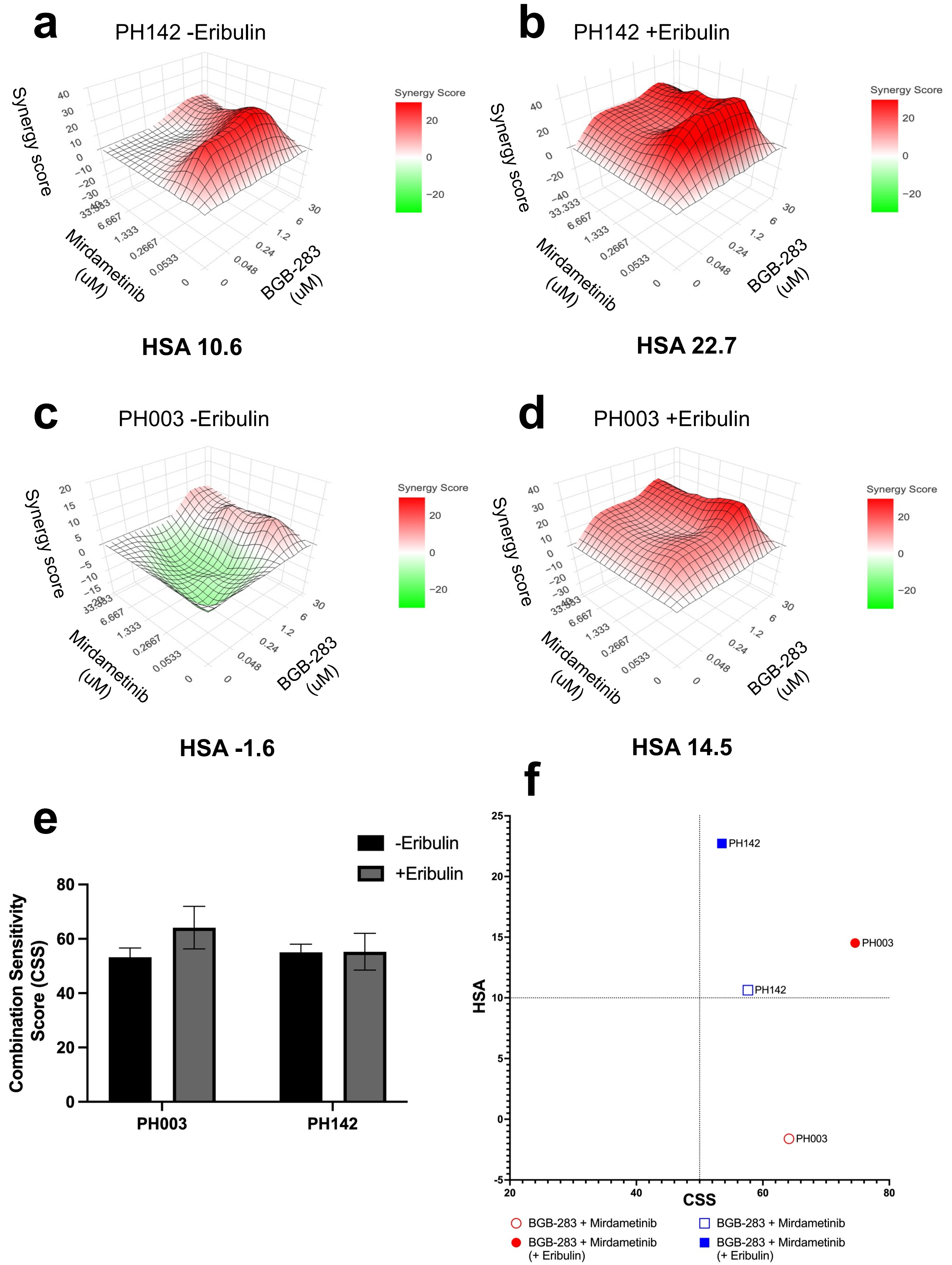

Supplement: Supplementary file 1 — Supplementary Material 1: Supplementary Figure S1: Drug screening of a mouse OCS cell line identifies sensitivity to independent cisplatin- and eribulin-based combinations. a) Dose response curves for eribulin and cisplatin in the mouse OCS cell line (left), validation of EC20 doses (right), and surviving fractions for chosen EC20 concentrations (bottom); b) Waterfall plot of cisplatin-based combination drug effects in the initial screen. DEffect values are calculated by subtracting the effect of the compound alone from the effect of the compound in combination with cisplatin. Most effective cisplatin-based combinations are indicated on the right as well as compounds selected for the validation screens (green); c) Waterfall plot of eribulin-based combination drug effects in the validation screen. DEffect values are calculated by subtracting the effect of the compound alone from the effect of the compound in combination with eribulin. Most effective eribulin-based combinations are indicated on the right as well as compounds selected for the validation screens (green). SF, survival fraction. Supplementary Figure S2: Validation of EMT-related single agent hits from the drug screen in cell lines. a) Dose response curves for each compound in four cell lines (two OCS and two HGSOC); b) EC50 values for each of the EMT-related single agents tested in cell lines; c) Heatmap of EC50 values for each EMT-related single agent in each cell line; d) Average EC50 values for the HGSOC cell lines combined compared to OCS cell lines combined. Supplementary Figure S3: Validation of N-MYC-related single agent hits from the drug screen in cell lines. a) Dose response curves for each compound in four OCS cell lines; b) EC50 values for each of the N-MYC-related single agents tested in cell lines; c) Heatmap of EC50 values for each N-MYC-related single agent in each cell line. Supplementary Figure S4: Validation of all single agent hits from the drug screen in organoids. a) Dose response cu [file 13046_2025_3629_MOESM1_ESM.pdf]
